# Frequency chirped Fourier-Transform spectroscopy

**DOI:** 10.1038/s42005-023-01157-5

**Published:** 2023-03-23

**Authors:** Sergej Markmann, Martin Franckié, Mathieu Bertrand, Mehran Shahmohammadi, Andres Forrer, Pierre Jouy, Mattias Beck, Jérôme Faist, Giacomo Scalari

**Affiliations:** Institute for Quantum Electronics, ETH Zürich, Auguste-Piccard-Hof 1, Zürich, 8093 Zürich, Switzerland

**Keywords:** Optical spectroscopy, Optical materials and structures

## Abstract

Fast (sub-second) spectroscopy with high spectral resolution is of vital importance for revealing quantum chemistry kinetics of complex chemical and biological reactions. Fourier transform (FT) spectrometers can achieve high spectral resolution and operate at hundreds of ms time scales in rapid-scan mode. However, the linear translation of a scanning mirror imposes stringent time-resolution limitations to these systems, which makes simultaneous high spectral and temporal resolution very difficult. Here, we demonstrate an FT spectrometer whose operational principle is based on continuous rotational motion of the scanning mirror, effectively decoupling the spectral resolution from the temporal one. Furthermore, we show that such rotational FT spectrometer can perform Mid-IR dual-comb spectroscopy with a single comb source, since the Doppler-shifted version of the comb serves as the second comb. In our realization, we combine the advantages of dual-comb and FT spectroscopy using a single quantum cascade laser frequency comb emitting at 8.2 μm as a light source. Our technique does not require any diffractive or dispersive optical elements and hence preserve the Jacquinot’s-, Fellgett’s-, and Connes’-advantages of FT spectrometers. By integrating mulitple broadband sources, such system could pave the way for applications where high speed, large optical bandwidth, and high spectral resolution are desired.

## Abstract (web summary)

Fast and high-resolution Fourier transform spectrometers are indispensable for cutting-edge infrared spectroscopy. In this study, the authors employed a newly-designed fast-rotating retroreflective, broadband delay line demonstrating fast dual-comb spectroscopy with a single mid-infrared optical comb from a quantum cascade laser emitting at 8 micrometers.

## Introduction

Real-time monitoring of non-repetitive events is currently a rapidly developing area in the field of instrumentation^1^. Rapid optical analysis in applications such as chemical reaction monitoring^2^, protein dynamics^3^, micromachining^4^ or phase transitions in novel material systems^5^ is of uttermost importance to understand the underlying complex dynamics. A general purpose instrument for spectroscopic characterisation is a Fourier-Transform (FT) spectrometer. However, the scan rate of the traditional FT spectrometer is often too slow when compared to the timescale of the physical processes of interest^6^. This is due to the fact that the information is acquired via the linear displacement of a mirror, which results in a trade-off between the temporal and spectral resolutions given that the system is not limited by the signal-to-noise ratio. In the last two decades two different directions appeared in the technological development of spectrometers. On one hand, the miniaturisation of spectrometers^7^ which is very promising for cost-effective lab-on-a-chip systems^7–12^ for specialized industrial applications. On the other hand, several new operational principles have been proposed and demonstrated with the goal of eliminating the above-described disadvantages of traditional benchtop FT spectrometers. Recently, successful proof-of-concept of phase-controlled FT spectroscopy^13^ and time-stretch spectroscopy^14–17^ have been demonstrated, which outperform the acquisition speed of traditional FT spectrometer by several orders of magnitude, while maintaining a relatively high spectral resolution. However, these newly developed methods, operating also in the Mid-IR, rely on dispersive elements such as gratings, dispersive fibers, lenses or prisms. Hence, they can be operated efficiently and continuously only over a limited optical bandwidth, where one has to sacrifice the spectral resolution at the cost of spectral bandwidth. An ideal instrument would cover a wide bandwidth, from optical down to THz frequencies. Although the optical and infrared spectral regions are of interest for certain applications, the most relevant one for molecular spectroscopy is the so-called molecular fingerprint region which covers mid-infrared (mid-IR) wavelengths from 4 to 12 μm. Recent progress in dual-comb spectrometers with mid-IR quantum cascade lasers (QCLs)^18^ have made that approach extremely attractive. Such a system acquires information not only on microsecond times scales, but can also be used for high-resolution spectroscopy via spectral interleaving^19^ with high brightness sources, which is very important for highly absorbing samples. However, there is a fundamental issue, which is the requirement of coherence for both frequency combs. The lack of mutual coherence of two free running lasers results in additive noise and the absence of an absolute frequency reference^20^. The latter can be overcome, as has been recently shown in^21^, at the cost of additional spectrometer complexity. These technological hurdles raise the question of whether it is possible to perform dual-comb spectroscopy with a single frequency comb source, without sacrificing spectral resolution and still keeping an acquisition speed sufficient for the majority of high-speed applications (typically 0.1–100’s of ms).

This question naturally leads one to consider the incorporation of the frequency comb source into a FT spectrometer with the goal of preserving the advantages of a dual-comb system and the flexibility of the FT spectrometer. Such integration has been realized in Ref. ^22^. Even though the comb modes (mode spacing of 140 MHz) were not resolved in this work due to the limitation of the mechanical delay line (resolution of 1.5 GHz), and the interferogram acquisition was only slightly below 5 min, it demonstrated the high potential of this approach. In ref. ^23^ a surpassing of the optical path-limited resolution was demonstrated where a frequency comb was used as a source in the FT spectrometer. However, this system was limited by the acquisition speed of the FT spectrometer. To address the growing demand for real-time spectroscopy in the NIR and mid-IR (2–14 μm), the temporal and spectral performance of existing FT spectrometers has to be improved. Several optical delay line systems have been developed which are based on a rotational optical element, such as a circular involute stage^24^ and curvilinear reflectors^25,26^. These two systems have in common that the optical path difference is angle-dependent, obtained via rotational optics which guide the incoming beam to a static reflector which reflects the beam back. Hence, in such systems the incoming and out going beam are anti-parallel, which will result in large undesired feedback into the laser source. Moreover, these systems do not support transversally extended light beams and hence possess a phase-dependent-delay across the beam diameter, which make them impractical for usage in the FT spectrometer. On the other hand, a compact flexible multi-pass rotary delay line using spinning micro-machined mirrors have been developed^27^. The big disadvantage of these systems is a relatively small achievable optical path difference, which is typically <1 mm, and the requirement of fiber optics and optical circulators, thus limiting this system to a narrow optical bandwidth. Another interesting approach was shown in ref. ^28^ where a rotating retro-reflector in combination with a static planar mirror is used. The incoming beam which is guided via a rotational retro-reflector to the plane mirror is back reflected. The disadvantage of this system lies in non-separable incoming and outgoing beams. Moreover, the system size grows linearly with the increase of the optical achievable delay path. This make the system unpractical for delay lengths of 3 cm and more and performance at large acquisition speeds.

By taking the advantages and disadvantages of the above described optical delay systems into account, we defined some design criteria that our system should fulfill: (I) Support of a large optical bandwidth; hence dispersive elements have to be avoided. (II) High spectral resolution; 0.5 cm^−1^ or lower. (III) Preserved polarisation of the light source. (IV) Fast acquisition speeds: 0.1–100’s of ms. (V) Easy integration into existing systems and hence small footprint. (VI) Decoupled temporal and spectral resolution as well as the decoupling of the incoming and outgoing beams. (VII) Support of extended beam diameter size.

In this work we demonstrate an FT spectrometer based on a rotational delay line and a single frequency comb light source, which fulfills the conditions listed above and overcomes many of the difficulties of dual-comb and linear FT spectroscopy. In conventional FT spectrometers the direction of the moving mirror has to be reversed. i.e. the center of mass of the mirror undergoes translational motion with inversion points. This sets the maximum achievable acquisition speed for a given spectral resolution, due to the mirror inertia which would require large external forces that scale quadratically with the acquisition time. The use of a rotational element decouples the spectral resolution from the interferogram acquisition time. This is due to the fact, that the spectral resolution is fixed and is given by the total optical path length of the rotational delay line. The temporal resolution is proportional to the angular velocity of the rotational optical element and is independent on the total optical path length. With our rotational FT spectrometers we can reach ms acquisition speeds which can be further improved by technological developments as will be discussed later. Such a high acquisition speed results in a high signal-to-noise-ratio (SNR), due to minimization of the 1/f noise within the signal bandwidth; such dependence has been observed to reduce the noise by two orders of magnitude moving from Hz to kHz for a free running mid-IR QCL (comb)^29,30^. The combination of our rotational FT spectrometer with the frequency comb as a light source does not limit our system in spectral resolution given by the total optical path length. As a proof-of-principle, by exploiting a quantum cascade laser frequency comb as a light source, we demonstrate high spectral resolution measurements leveraging on the interleaving technique already employed in dual-comb spectrometers^31,32^. We could resolve the transmission resonances of a silicon etalon and the absorption lines of a Doppler-broadened low-pressure methane gas sample (CH_4_) with a resolution < 250 MHz. Moreover, we demonstrate simultaneous high resolution and fast acquisition capabilities by monitoring the absorbance lines of low pressure methane during the evacuation of a gas cell. It has to be underlined that our approach uses only reflective elements which enable us in principle to cover continuously a frequency band from optical down to THz frequencies with almost always above 90% reflectivity.

## Results

### Rotational FT-spectrometer

**Fig. 1:**
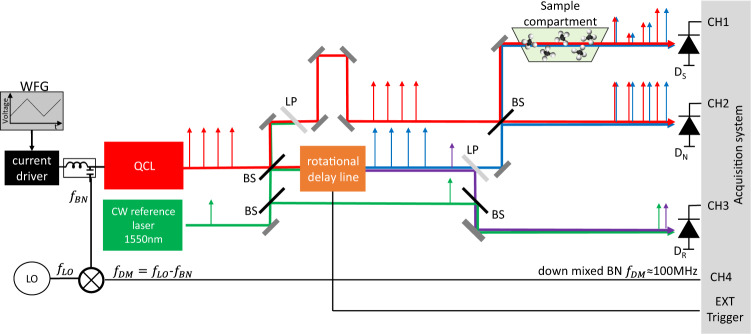
Block scheme of the rotational FT spectrometer with a Quantum Cascade Laser. A CW reference laser which is used to provide a frequency reference and serves also as a tool for mapping out and removing the induced frequency chirp imposed by the nonlinear rotational delay line. QCL and CW laser beam are spatially superimposed via a beam splitter (BS) and also separated via the optical low-pass filter (LP). Interferograms of the QCL are acquired with the sample (D_*S*_) and normalization (D_*N*_) detector, where the reference interferogram of the CW laser is recorded with detector D_*R*_. All measurements are synchronized with the trigger from the rotational delay line. The function generator (FWG) can be used for continuously tuning of the QCL operation point by applying a ramp to the current driver of the QCL. The inter-modal beat note signal can be extracted directly from the waveguide via bias-tee and the down-mixed version of it *f*_*DM*_ can be recorded with the help of the local oscillator LO.

**Fig. 2:**
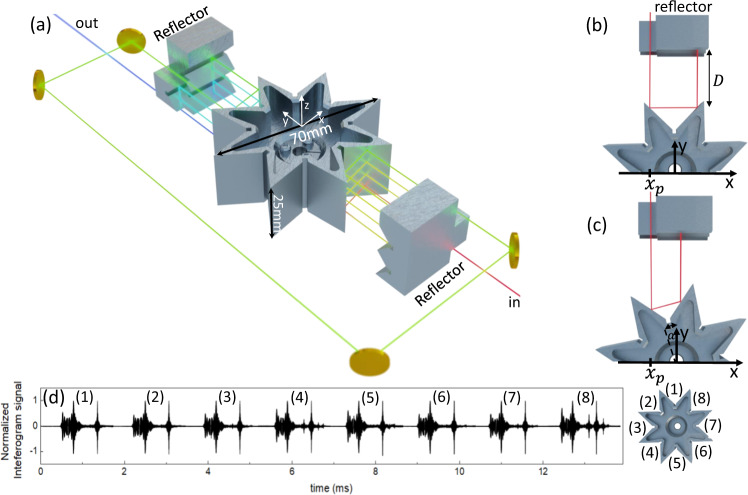
Rotational delay line. **a** Realization of the rotational delay line (RDL) consisting out of three-dimensional rotational retro-reflecting (RR) octagrammic prism and a static retro-reflecting system (SR). The SR is arrange in such a way with respect to RR, that multiple reflections between SR and RR are possible. By exploiting the point symmetry of the system, the optical path delay can be doubled with two SR on opposite sides of the RR. **b**, **c** Top view of a beam propagation in the RDL at rotation angle 0^∘^ and *α*. **d** Recorded interferogram on each rotational retro-reflector of delay line.

A diagram of the rotational-based FT spectrometer is shown in Fig. 1. In this first demonstration we employ a mid-IR QCL frequency comb^18^ as a light source emitting around 8 μm wavelength. However, any collimated light source with beam diameter below 7 mm could be used in our spectrometer. The QCL is a 2.5 mm-long ridge (free spectral range of ≈ 19.7 GHz) with high-reflection coating on the back facet and with maximum emission power of 60 mW at *T* = −20 °C. In contrast to amplitude-modulated frequency comb light sources, which emit light pulses spaced by the cavity round-trip time (*t*_*rep*_ = $\frac{1}{{f}_{rep}}$), the QCL emits a frequency modulated comb (FM-comb)^33,34^. This type of a frequency comb exhibits a quadratic dispersion of the phases of the longitudinal laser modes, resulting in a linearly chirped instantaneous frequency with nearly constant intensity^35^. The absolute frequencies *f*_*m*_ of the comb teeth in the optical domain are described by *f*_*m*_ = *f*_*ceo*_ + *mf*_*rep*_, where *f*_*ceo*_ is the carrier envelope offset frequency, *f*_*rep*_ the repetition rate and *m* an integer representing the mode number. The emitted and collimated QCL light beam is divided by a beam splitter (70/30) into two paths; one static beam path and one which passes through the rotational delay line (RDL). The two beams are then recombined on a second beam splitter (50/50), creating a sample and a normalization beam path. Since the beam is going through the RDL, it acquires an optical path delay (OPD) with respect to the static beam, and two separate interferograms can be recorded at the sample (*D*_*S*_) and normalization (*D*_*N*_) detectors, respectively. We use thermo-electrically cooled HgCdTe detectors from Vigo system (model:PVMI-4TE-10.6-1x1-MIP-10k-100M-TO8-wZnSeAR) with a peak sensitivity at 8 μm and 100 MHz bandwidth (the response of the detectors with the employed system’s power is shown in Fig. S2). As for dual-comb spectroscopy setups, the normalization detector is required for canceling common-mode fluctuations which can originate from power and frequency instabilities of the QCL comb. The key element of our setup is the RDL which is schematically shown in Fig. 2a. It consists of a rotational retro-reflecting (RR) octagrammic prism and a static retro-reflecting (SR) system, which are arranged so that the incoming beam towards the SR is redirected along the rotation axis (z-axis) and reflected back towards the RR. The RR can in principle consist of any number *M*≥2 retro-reflectors, symmetrically arranged around the rotational axis z. The optimal *M* will depend on the repetition rate of the laser, the speed of the motor, the tolerable losses due to number of reflection in the delay line, and the desired acquisition rate. In our case, *M* = 8 is optimal for the target of recording a single IFG in ~ 1 ms or faster. The SR consists of two retro reflectors arranged next to each other and shifted along the z-axis, so that the incoming beam is reflected several times at different heights of the RR. In this configuration, the incoming and out-going beams on the same side of the RR have opposite propagation directions and are separated in height. Moreover, the retro-reflecting arrangement preserves the polarisation of the laser beam. The outgoing beam can be guided via mirrors to the opposing side of the RR in order to bring it to the height of the initial incoming beam. This way of lowering the beam has the additional advantage of doubling the optical path difference, which allows for resolving the comb modes for our 2.5 mm laser. Figure 2b, c illustrate the beam propagation at zero degree and at an angle *α*. The general expression for the total optical path length in the RDL is given by (see Methods for the detailed derivation):

1 $$L(\alpha )= \, 	2N\left(\right.D+{S}_{1}{x}_{p}-{C}_{1}+\sqrt{{(T-{x}_{p})}^{2}+{({S}_{3}T+{C}_{3}-{S}_{1}{x}_{p}-{C}_{1})}^{2}}\\ 	 -\,{S}_{3}T-{C}_{3}\left.\right)+2(N-1){l}_{4},$$

where ${S}_{1}=\tan (\alpha -\pi /4)$, ${C}_{1}=\cos (\alpha )b+{S}_{1}\sin (\alpha )b$, ${S}_{2}=\tan (\alpha +\pi /4)$, ${C}_{2}=\cos (\alpha )b+{S}_{2}\sin (\alpha )b$, ${S}_{3}=\tan (2\alpha )$ and *C*_3_ = *C*_1_ + *S*_1_*x*_*p*_ − *S*_3_*x*_*p*_. Here, *α*(*t*) = *ωt* is the rotation angle, *ω* the angular velocity of the RR, *t* is the laboratory time, and *N* is the number of times the beam is injected onto the RR (in our case *N* = 4). The derivation of Eq. (23) can be found in the Methods section. The OPD is then Δ*L* = *L*(*α*) − *L*(*α* = 0). Figure 3a shows the optical path length according to Eq. (23) when the incoming beam enters the RDL at *x* = *x*_*p*_ and at $x={x}_{p}+\frac{d}{2}$ at *α*(*t* = 0) = 0, where *d* is the beam diameter. It shows that the incoming position of the beam on the *x*-axis (Fig. 2b, c) determines the maximum achievable OPD. It is important to note, that the phase delay is independent on the laser beam diameter *d*, which is crucial for spectroscopy applications. The beam diameter size only influences a maximum achievable OPD. The maximum achievable OPD is given for *d* = 0 and the entry point *x* = *x*_*p*_. Moreover, the phase delay is nonlinear in the angle *α*, and hence in the laboratory time t.

**Fig. 3:**
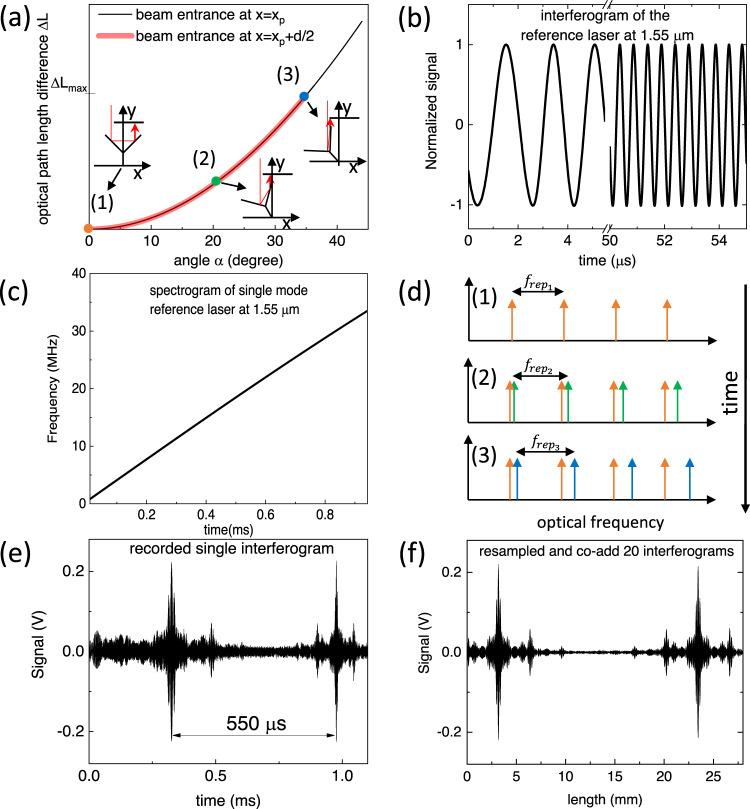
Operational principle of the rotational spectrometer. **a** Induced nonlinear optical path length difference ΔL by rotational delay line as a function of rotation angle *α*. The maximal achievable path length difference depends on the entry point of the light beam along the x-axis (Fig. 2b, c). The orientation of the rotational delay line with respect to the incoming beam is shown for the selected point. **b** Interferogram time slices of the continuous wave (CW) reference laser on detector D_*R*_. **c** Spectrogram of CW reference laser which shows almost linear induced frequency chirp by rotational delay line. **d** Illustration of induced frequency chirp to a frequency comb. Three different color coded time points of comb spectrum, which corresponds to the same color-coded points on the curve in **a**. **e** Recorded quantum cascade laser (QCL) interferogram on one of eight retro-reflectors of the octogram. Increasing frequency chirp is clearly visible with increasing time. **f** Resampled QCL inteferogram of **e** on zero crossings of a CW reference laser from **b** with a coherently co-added 20 consecutive interferograms.

In order to take the nonlinear OPD into account, as well as to provide an absolute frequency reference for the QCL spectrum, a single mode continuous-wave (CW) reference laser (in our case we used part of the signal from a Rio GRANDE 1550 nm High Power Laser Module) is spatially superimposed on the QCL beam. In this way, the two beams are co-propagating through the RDL and experience the same nonlinear OPD. After propagation through the RDL the reference beam is spatially separated from the QCL one by means of an optical low-pass filter, which acts as a reflective element for the reference laser and transmissive for mid-IR QCL beam. As in the case of the QCL, a static and a non-static CW reference laser beam are combined onto a beam splitter and the resulting interferogram is recorded on the reference detector *D*_*r*_. More details on the constraints and the alignment of the spectrometer are given in the Methods section.

In addition to the detection of the optical signals, we exploit the fact that the beating of the linearly spaced frequency comb modes of the QCL can be directly measured in the radio-frequency domain. The beating gives rise to a modulation of the laser bias^18^ and can be extracted from the device through a bias-tee (Fig. 1). We record this inter-modal beat note signal by down-mixing (DM) it with a local oscillator LO with the frequency *f*_*LO*_ to the frequency *f*_*DM*_ ≈ 100 MHz. All signals are then digitized with the acquisition card (model: Spectrum M4i.445x-x4) with 500 MS/s and 14 bit resolution. The digitized data is then band-pass filtered with the goal of suppressing the noise outside the signal bandwidth. With the help of the LO and the recorded *f*_*DM*_ signal, the repetition rate *f*_*rep*_ = ∣*f*_*LO*_ − *f*_*DM*_∣ of the QCL is determined. The optical frequency of the reference CW laser was determined with precision better than few 100s of MHz using an optical spectrum analyser. This reference laser features frequency stability of <15 kHz. Since the zero crossings of the CW reference interferogram are used for resampling the QCL interferogram, the frequency accuracy of the resampled QCL spectrum is limited by the optical frequency resolution of the CW reference laser which is several order of magnitudes larger than the laser stability of <15 kHz. However, the frequency of the reference laser can be calibrated more precisely (several MHz) with a narrow absorption line of a known reference gas, as also realized in this work in the interleaving section. It is worth noting that a laser with a frequency stabilty of 1–2 MHz would be also sufficient for our experiment since we perform a calibration on narrow absorption line. The developed RDL provide around 3 cm of OPD. A maximal RR angular frequency of 85 Hz (limited by the employed motor’s stability) results in relatively high acquisition rates, with a duty cycle of 50% as shown in Fig. 2d, where each interferogram is recorded on one of the retro-reflectors of the octogram. Moreover, as we will discuss later, any imperfections in the fabrication of the RR might result in tiny differences in the interferogram acquired on each retro (as can be seen in Fig. 2d (1) and (8)). However, since a normalization detector is used, the important measure is the spectral ratio of the sample and the normalization detector which will eliminate any fabrication imperfections of the RR.

### Operational principle of the rotational FT spectrometer

Since the individual interferogram is acquired within a ms or even faster, the corresponding linear speed of the mirror makes the Doppler effect^36^ relevant. The relativistic Doppler effect shifts the frequency *f*_*m*_ of an electromagnetic wave to the frequency ${f}_{{D}_{m}}$ according to

2 $${f}_{{D}_{m}}={f}_{m}\sqrt{\frac{1-v/c}{1+v/c}}\approx {f}_{m}(1-v/c),$$

where $v=\frac{d{L}_{tot}}{dt} < < c$ is the rate of change of the optical path length *L*_*tot*_ and *c* is the speed of light. The frequency shift depends on the rotational speed of the motor and the OPD which is given by the radius of the rotating octogram, and the number of reflections onto the rotating octogram (see Methods), which in our case gives an average *v* of about 40 m/s. The Doppler effect of mid-IR frequencies at these speeds is significant and amounts to several MHz. Since the Doppler-shifted and non-Doppler shifted light beams are superimposed onto a detector, the neighboring optical frequencies of the beams will beat and generate an heterodyne signal in the radio frequency (RF) domain given by

3 $${f}_{R{F}_{m}}(t)={f}_{m}\frac{1}{c}\frac{\,{{{{\rm{d}}}}}{L}_{{{{{\rm{tot}}}}}}(\alpha (t))}{{{\mbox{d}}}t}={f}_{m}\frac{\partial {L}_{{{{{\rm{tot}}}}}}(\alpha )}{\partial \alpha }\frac{\omega }{c}.$$

The frequency chirp induced by the nonlinear OPD Fig. 3a is visualised in Fig. 3b by recording an interferogram of the single-mode reference laser. Here, the first 5 μs of the recorded interferogram and the same time interval after 50 μs is shown, while the chirped Doppler shift of the entire interferogram is shown by the spectrogram in Fig. 3c.

In the case of a frequency comb source, the signal recorded on the detectors *D*_*S*_ and *D*_*N*_ without a sample is given by :

4 $$S=\Re \left(\mathop{\sum }\limits_{m=0}^{\infty }{A}_{m}\exp \left(\,{{\mbox{j}}}\,\left(2\pi {f}_{R{F}_{m}}(t)t+{\phi }_{m}\right)\right)\right)$$

where *A*_*m*_ is the amplitude and *ϕ*_*m*_ the phase of mode *m*. The nonlinear OPD will not only produce a chirp of all modes *m*, resulting in absolute frequency shifts of the mode frequencies ${f}_{R{F}_{m}}$, but will also directly modify *f*_*rep*_ of the comb during the interferogram acquisition. This effect is illustrated in Fig. 3d at three different time points, corresponding to the same color-coded points on the curve in Fig. 3a. The first orange point at *α*(*t* = 0) = 0 is related to Fig. 3d (1) and corresponds to the case with zero OPD and no Doppler shift. Increasing *α* to 20 degrees impacts the repetition rate of the comb (${f}_{re{p}_{2}}={f}_{re{p}_{1}}\cdot (1+v(\alpha =2{0}^{\circ })/c)$) as well as the absolute frequencies of the individual optical modes, which acquire a Doppler shift as well (Fig. 3d (2)). A further angle increase of the RR results in a larger Doppler shift and hence also larger ${f}_{re{p}_{3}}$ (Fig. 3d (3)). Such a frequency-chirped interferogram of a QCL frequency comb is shown in Fig. 3e (the visibility of the fringes for the QCL emitting single mode is reported in Fig. S3). In order to obtain a chirp-free interferogram from which the spectrum can be retrieved, the chirped QCL interferogram is resampled using the zero crossings of the reference laser interferogram. This is possible since both beams undergo identical nonlinear delay and the frequency response of the electronics is flat for the employed modulation speeds. The resampled interferogram of the QCL frequency comb with improved SNR by coherently averaging 20 interferograms, which averages out uncorrelated noise present in individual interferograms is shown in Fig. 3f. The resolution of the system given by the maximum OPD of 3 cm is 10 GHz (0.33 cm^−1^), while the single burst acquisition speed on one of the octogram retro-reflectors is 1 ms which is given by the maximum angular velocity of the motor and the geometrical realisation of the rotating and static retro-reflectors via Eq. (23).

### Spectroscopy with rotational FT spectrometer

In order to perform absorption spectroscopy on a sample, a sequence of two measurements is needed: a background spectrum and sample spectrum. The background spectrum is acquired on the sample *D*_*S*_ and normalization detector *D*_*N*_ without a sample present. Since the QCL spectrum is extracted from the acquired single-burst interferogram with the knowledge of the computed *f*_*rep*_, we employ sub-nominal resolution method described in ref. ^37^, in which precisely one repetition rate of the laser is acquired, in order to eliminate spectral leakage. As pointed out earlier, the normalization detector *D*_*N*_ is used to suppress common noise on the two beam paths, stemming from laser relative intensity noise (RIN). Please note, that the laser RIN of a free running QCL significantly reduces for acquisition speed of 1 ms per interferogram^30^. This is also where the speed of our rotational FT spectrometer plays an important role. Moreover, we have also verified that the light scattering due to surface quality (diamond polish aluminum with surface roughness <10 nm Ra, arithmetical mean deviation, see Fig. S1 in Supplementary Material) of the RDL (RR and RS) does not contribute to the intensity noise. This is verified by computing the background ratio:

5 $${R}_{BG}=\frac{{S}_{S}^{BG}}{{S}_{N}^{BG}},$$

where ${S}_{S}^{BG}$ and ${S}_{N}^{BG}$ are the spectra recorded by detector *D*_*S*_ and *D*_*N*_, respectively. Placing a sample into the sample beam path (Fig. 1) and repeating the spectral measurements, the sample transmittance can be computed as

6 $$T=\frac{{R}_{S}}{{R}_{BG}}$$

where

7 $${R}_{S}=\frac{{S}_{S}^{S}}{{S}_{N}^{S}}$$

and ${S}_{S}^{S}$ and ${S}_{N}^{S}$ are the spectra recorded with detector *D*_*S*_ and *D*_*N*_, respectively. We note that in the system configuration shown in Fig. 1 only the magnitude information, i.e. transmittance or absorbance, can be measured. However, arranging the spectrometer geometry as in the case for Dispersive FT spectroscopy^38^, where the sample is placed in one of the arms of the interferometer, the phase information could be obtained as well. Since in our case we utilize a normalisation detector for common intensity noise suppression of the QCL, a scheme as proposed by Schiller^39^ for dual-comb spectroscopy, where the second comb corresponds in our case to the Doppler shifted beam path, could be exploited as well.

### Amplitude noise of the system

**Fig. 4:**
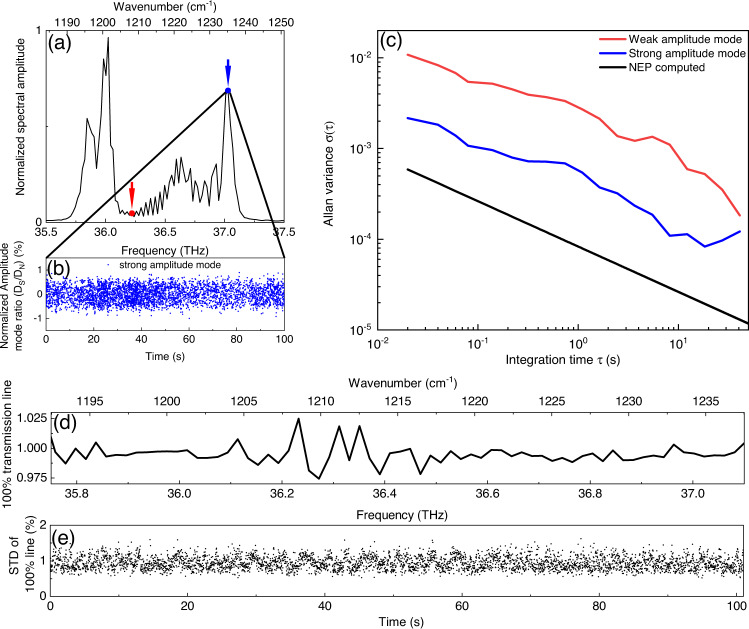
Amplitude noise investigation. **a** QCL comb spectrum on which Allan deviation is performed. Red arrow indicates reference frequency for the weak amplitude mode analysis. Blue arrow indicates reference frequency for the strong amplitude mode analysis. **b** Temporal evolution of normalized amplitude mode ratio of the strongest mode, which is marked with blue arrow in **a**. It shows the system stability over the time period of 100s with amplitude fluctuations below 1%. **c** Computed Allan variance of high (blue) and low (red) spectral amplitudes of the spectrum in **a** which is indicated with arrows in the same color code. The black curve shows the computed Allan variance taking the background (Noise equivalent power (NEP) of a detector) into account for a power of strong mode. **d** Background, or so called 100% line transmission, of a system for a single interferogram and **e** its temporal stability expressed as a standard deviation (STD) in % over the entire optical bandwidth over 100s.

Before demonstrating high-resolution spectroscopy we analyse the system sensitivity and discuss possible noise sources and their origin. The SNR of the system is given by the amplitude noise of the heterodyne beat note signal in the RF domain. For this purpose we analyse the amplitude noise fluctuations of the spectrum shown in Fig. 4a. Figure 4b shows a time trace of the normalized amplitude ratio of the strong mode, with an amplitude fluctuations < 1%, showing excellent stability over several minutes. To quantify the different noise sources affecting our spectrometer we compute the Allan deviation *σ*(*τ*) of comb teeth with high and low amplitudes, indicated in Fig. 4a with red and blue arrows. Figure 4c shows the Allan deviations as functions of the integration time *τ*. Additionally, we show the computed noise floor originating from the detector noise-equivalent power (NEP) computed from the spectrum from an high-intensity comb mode. Both the high and low amplitude peaks show that the noise of the system decreases with the square root of the integration time *τ* up to 40 s and the system SNR is close to the detector NEP, being larger by a factor of 2.8. This deviation might be originated from additional thermal and electrical noise sources which are not taken into account in the NEP evaluation. Thus, with the employed detectors and the laser, the SNR can be improved by longer integration times or by more powerful laser, since the detector saturation power is around 8 mW, whereby the average power on the detector is 0.5 mW. With higher power close to the saturation power we would expect improvement of the SNR by factor 10. At longer integration times (*τ* > 10 s) we can reach SNR of 10^4^ and 10^3^ for strong and weak modes, respectively. We would like to point out that the 1/f noise in the system is minimized, due to fast acquisition, since we observe a slope of − 1/2 for the entire Allan plot which is characteristic of NEP-noise-limited systems. In order to perform long-term measurements, we have to quantify the background stability. For this reason we record the system background on time scales of minutes. Figure 4d shows a normalized system background (*R*_*BG*_) spectrum, so called 100% transmission line, for a single interferogram over the entire comb bandwidth. Since the comb spectrum consist of two lobes with high power on the sides and with a low power region in the center of the spectrum, the computed 100% line also reflects this spectral power distribution with 2% deviation in the weak spectral region. For demonstration of the system and hence background stability we show the computed standard deviation (STD) of the 100% line over the entire spectrum in Fig. 4e. As we can see from Fig. 4e the system background remains stable over 100 s with deviations below 1.5%. This allows to perform long-term measurements which are characteristic of high resolution spectroscopy via interleaving technique^32^.

### Interleaving spectroscopy

#### Interleaving on silicon etalon

**Fig. 5:**
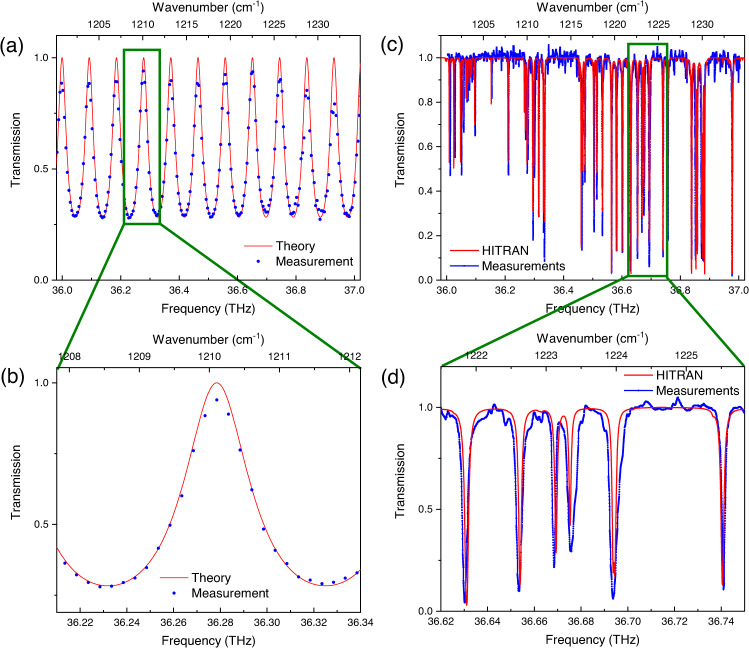
High resolution spectroscopy. **a** Measured interleaving spectrum (blue dots, error bars smaller than the dots) of ≈ 500 μm thick silicon etalon within 7 s with a binned frequency resolution down to 5 GHz. Theoretical computed etalon transmission spectrum (red line). **b** Zoom in into (**a**). **c** Doppler broaden methane (CH_4_) spectrum recorded via interleaving (blue dots) at a pressure of 200 mbar within 25 s with frequency binned resolution down to 250 MHz and the HITRAN data base reference (red line). **d** Zoom in into (**c**) with sub-GHz resolved methane absorption lines.

In order to demonstrate the system capabilities for high-resolution spectroscopy, we use the spectral interleaving technique, similar to what described in Ref. ^32^. In the case of interleaving, the resulting point spacing resolution is smaller than the free spectral range of the laser given by the QCL cavity length, which in our case corresponds to a resolution of ≈ 19.7 GHz. In contrast to dual-comb spectroscopy, where two synchronised current ramps with different slopes are required for the laser tuning^32^, we apply a single triangular current modulation ramp (Fig. 1) to the QCL current driver (Wavelength electronics QCL1000) which is controlled by means of a wave function generator. The interferogram acquisition trigger (the Hall sensor of the motor) does not need to be synchronized with the function generator, since the extracted frequencies from individual spectra can be sorted in post-processing. The triangular current ramp of the function generator is set so that the overall QCL spectrum can be tuned by one *f*_*rep*_. In this way, we can access frequencies between modes and even provide a gap-less spectral coverage over the entire optical bandwidth of the QCL comb. Firstly, we demonstrate the high-resolution capability of the system on ≈ 500 μm thick silicon etalon. For this measurement, we electrically tune the QCL spectrum over one *f*_*rep*_ in about 7s . During this time, the measurements are acquired continuously at a rate of 50 Hz. Due to the slow current ramp rate and fast interferogram acquisition rate (1 ms) with 20 ms periodicity (acquisition on the same single retro-reflector), the information within the recorded single-burst interferogram is not influenced by the slow current changes induced by the current ramp. As explained earlier, we record a background and a sample measurement, and extract information from a single-burst interferogram via the sub-nominal resolution technique^37^. The corresponding interleaved data is shown in Fig. 5a, where 20 consecutive interferograms are coherently co-added before a spectrum is computed. The resulting spectra are then frequency binned to 5 GHz resolution. As it can be seen in Fig. 5a and the zoomed region (Fig. 5b) the recorded interleaved spectra fit well the computed etalon spectrum. Measurements deviation from the model, especially at frequencies with high transmission can be attributed to losses in the silicon etalon which were not taken into account in the model. Also small angle deviation from perpendicular incidence on the etalon will result in deviation from the model. This measurement shows that in a relatively short time period of 7 s, we can clearly resolve etalon features with resolution < 0.15 cm ^−1^. This addresses the question whether sub-GHz spectroscopy is possible with our spectrometer.

#### Interleaving on methane

A more challenging and practically relevant scenario is high-resolution spectroscopy on low-pressure Doppler-broadened methane lines. For this experiment, the background measurements were carried out with a 10 cm-long evacuated gas cell inserted into the sample beam path and the sample measurements were performed on the filled gas cell with CH_4_ at a pressure of 200 hPa. Contrary to the low-finesse etalon in the previous section, the low pressure methane lines have sub-GHz linewidths. The high-resolution measurements were performed in the same way as for the etalon, but with the QCL current ramp period set to 25 s. In this way, thousands of interferograms are recorded, whereby each interferogram is recorded on ms time scale, and 10 consecutive ones are co-added before computing each spectra. With such a slow current ramp period we can ensure a high frequency resolution and resolve low pressure Doppler-broadened methane lines. The resulting interleaved spectrum of methane is shown in Fig. 5c, where the resolution has been reduced by frequency binning to 250 MHz. There are two reasons for frequency binning to 250 MHz. First, it results in improved SNR and second the interleaved spectrum is equidistant. A zoom into the spectrum of Fig. 5c is displayed in Fig. 5d and shows that the system is capable of resolving Doppler-broadened molecular absorption lines with sub-GHz linewidths. However, the resolved linewidths of some methane lines are broadened by up to 5% compared to the bare HITRAN 2020 data base spectrum^40^. For the computed HITRAN data spectrum no instrumental linewidth has been taken into account, because we do not expect any line broadening from our rotational FT spectrometer in combination with the QCL frequency comb. Additionally, we observe that the 100% transmission line is not entirely flat. This measurement deviations can be explained with contributions from two different origins. Firstly, the QCL comb short-term stability can be affected by back-reflections in the system. Secondly, high-frequency electrical instabilities of the laser can occur while interleaving, since it is challenging to identify a current range where the QCL tunes continuously over one *f*_rep_ staying continuously in the comb regime. This will result in slightly different QCL states for background and sample measurement. We note that this noise source could be further reduced by using low-noise current sources and a more stable comb source. The broadening of some absorption lines with respect to the HITRAN database can have several origins. Spectral leakage occurs if the source laser is not a perfect comb over its whole bandwidth. We can notice this effect as a smooth roll-off on each side of the spectrum in Fig. 4a. During interleaving a current ramp is applied to the laser, which reduces laser stability and hence increases spectral leakage. Consequently, in the situation where a neighboring line tunes into an absorption line, the intensity of a specific line will also be modulated. This could explain the broad features observed on the 2nd and 3rd lines from the right in Fig. 4a, as well as the narrow features of higher than unity transmission throughout the spectrum, occurring at multiples of one *f*_rep_ from the methane absorption lines. Finally, the pressure gauge has an uncertainty of 10 mbar, which could explain a small part of the difference in linewidth. However, Fig. 5d shows clearly that all sub-GHz CH_4_ absorption lines can be well resolved. We demonstrated that sub-GHz spectral resolution displays a technical advantage of our system with respect to a typical state-of-the-art FT spectrometer, which achieves a resolution of 0.06 cm^−1^ (1.8 GHz) with a much larger footprint. More importantly, we demostrated millisecond acquisition times ms with 0.5 cm^−1^ (15 GHz) spectral resolution that is impossible to achieve for rapid scan commercial FT spectrometers. The typical spectral resolution for 10 ms acquisition time would be >25 cm^−1^, insufficient to resolve the laser modes.

### Time-resolved measurements on methane

**Fig. 6:**
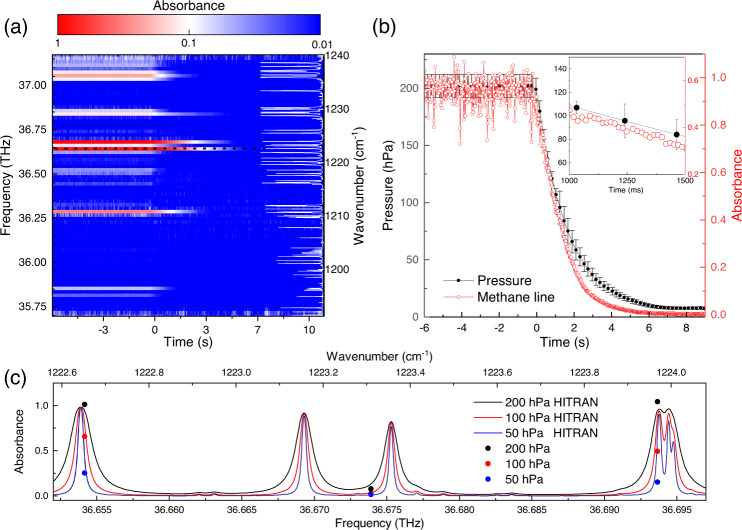
Time-resolved spectroscopy on methane. **a** Absorbance is computed from time resolved-transmission measurements on the filled gas cell to a pressure of 200 hPa which is evacuated via a pump. The computed methane absorbance for a pressure of 200 hPa is shown on the right side with white lines. Temporal evolution is monitored by initializing the evacuation of the gas cell a *t* = 0 s. The acquisition is performed with periodicity of 10 ms in the comb regime at a constant current mode. Simultaneously several low pressure methane lines are observed for *t* < 0 s. **b** Visualization of the temporal evolution of the strongest absorption line (marked with a dotted black line) in **a** and the corresponding pressure of the gas cell (error bars are the pressure gauge error). A zoom in is shown in the top right corner with 500 ms time duration starting 1s after opening the valve. **c** Computed HITRAN absorbance of methane at a pressure of 200 hPa, 100 hPa and 50 hPa and the measured absorbance close to the strongest absorbance peak at these pressure and hence corresponding laboratory time.

Replacing the translational delay stage used in state-of-the-art FTIR spectrometers by our rotational delay line enables much higher acquisition speeds for a given spectral point spacing. In order to demonstrate this capability of our system, we perform time-resolved spectroscopy on methane. We monitor multiple methane absorption lines simultaneously while reducing the pressure of the methane in the cell over a few (<6) seconds. Initially, a gas cell is filled with methane at a low pressure of 200 hPa via a gas inlet attached to a pump via mechanical valve, while the pressure is monitored with a vacuum gauge (Pfeiffer vacuum gauge controller TPG 361) in 100 ms time steps via a second connection. At *t* = 0 the valve is opened while simultaneously the pressure and the absorbance are monitored. The absorbance spectra are acquired in ~10 ms intervals by acquiring single interferograms in 625 μs with the QCL operated at a constant current in the comb regime. From the recorded transmittance (*I*/*I*_0_,) and the background measurement (empty cell), we compute the absorbance

8 $$A=-{\log }_{10}(I/{I}_{0}),$$

which is shown in Fig. 6a. For *t* < 0 s, where the valve is closed, we observe 10 methane absorption lines within the optical bandwidth of the comb. By opening the valve (*t* = 0 s) we observe a rapid absorbance decrease within 2 s. The absorbance is given by Beer-Lambert’s law as

9 $$A={\epsilon }_{\lambda }\cdot \tilde{c}\cdot l,$$

where *ϵ*_*λ*_ is the molar attenuation coefficient at wavelength *λ*, $\tilde{c}$ the amount concentration, and *l* the length of the gas cell. Both *ϵ*_*λ*_ and $\tilde{c}$ are functions of the gas pressure *p* and temperature. Since the linewidth of methane absorption lines (sub-GHz) at this low pressure is much smaller than the spectral point spacing of ~19.7 GHz of the particular QCL comb used in this experiment, the absorbance is measured off the center of the absorption lines. This results in a non-trivial time-dependence of the absorbance as the molar attenuation coefficient is both wavelength and pressure dependent; namely, a linewidth narrowing of the Doppler-broadened methane absorption lines is expected and hence a time-dependent change of *ϵ*_*λ*_, as well as a decrease in the concentration *c*(*p*). We demonstrate this effect by showing a cut of Fig. 6a (cut along the marked dotted line) along the temporal-evolution of the strongest absorbance line in Fig. 6b, and compare it to the monitored pressure inside the gas cell. The absorbance and the pressure decrease at the same rate down to a pressure of about 100 hPa. Moreover, for *t* > 6 s, which corresponds to a pressure of 7 hPa, the absorbance is zero, which can be explained by the fact that the QCL mode is either off from the resonance of the methane absorbance line, or it is located in the absorption tail, thus resulting in a weak absorption. We show the pressure related linewidth narrowing of methane in Fig. 6c where we compute the methane absorbance exemplary for three different pressures and overlap the measured absorbance points which corresponds to measurements times when these pressure are reached. We observe a good agreement of spectral and temporal resolved methane absorbance lines. Please note, that each absorbance line can be seen as an independent barometer, hence by calibrating each line one can increase the accuracy of the measurement. Our spectrometer demonstrates high temporal and spectral resolution which is predominant over the state-of-the-art FT spectrometers, which are not able to provide such simultaneous temporal and spectral resolution.

## Conclusion

In this work we have demonstrated a proof-of-principle rotational FT spectrometer which decouples the spectral resolution from the temporal one. Dual-comb spectroscopy with acquisition speeds in the range of ms is performed using a single QCL frequency comb. A clear benefit of a fast acquisition is the reduction of the 1/f noise within the signal bandwidth, which is inevitable in the state-of-the-art FTIRs operating at lower speeds. This is due to the fact, that the RIN of a free running QC laser is reduced at least by two to three orders of magnitude moving from the 100’s Hz region to the >10 kHz region^29^. Additionally, we have demonstrated high resolution, broadband spectroscopy capabilities on a low-finesse etalon and low-pressure, Doppler-broadened methane lines via the interleaving technique. Furthermore, since the geometry of the rotational delay line allows incorporation of multiple sources, either via time-multiplexing scheme or under simultaneous operation, a broader optical bandwidth could be achieved with minimal modifications to the setup. We would like to point out that our developed system may be exploited for the recently proposed frequency comb ptychoscopy scheme^36^, where an external signal is analysed with a frequency comb and a Doppler-shifted version of it. Since the frequency shift of the Doppler shifted comb can be adjusted to acoustical frequencies (sub MHz), the system can also be employed for photo-acoustic dual-comb spectroscopy^41^. The geometrical layout of the rotational delay line can be attractive for the implementation of multidimensional FT spectroscopy^42^ with a single frequency comb source. The compact system demonstrated in this work can be of high interest for performing high spectral resolution and time-resolved spectroscopy, especially if ultra broad band white, NIR and mid-IR supercontinuum laser sources are to be employed^43–46^. Moreover, in the case of light sources with stable temporal intensity, the complexity of the system can be reduces by eliminating the normalization detector and reducing the acquisition system to two channels. The nominal spectral resolution can be significantly improved by a factor 2 to 4 by increasing the size of the RDL or by increasing the number of reflections (*N* in Eqn. (23)). An increased temporal resolution is also readily achievable by replacing the existing motor with a more performing one. This would increase the single-spectrum acquisition speed up to 30 kHz (33 μs), which is sufficient for the majority of spectroscopy applications, such as reaction monitoring^47^, leakage monitoring^48^ and in-line process analytical technology (PAT)^49^.

## Methods

### Derivation of the angle dependent path length of the rotational delay line with independent beam diameter phase delay

**Fig. 7:**
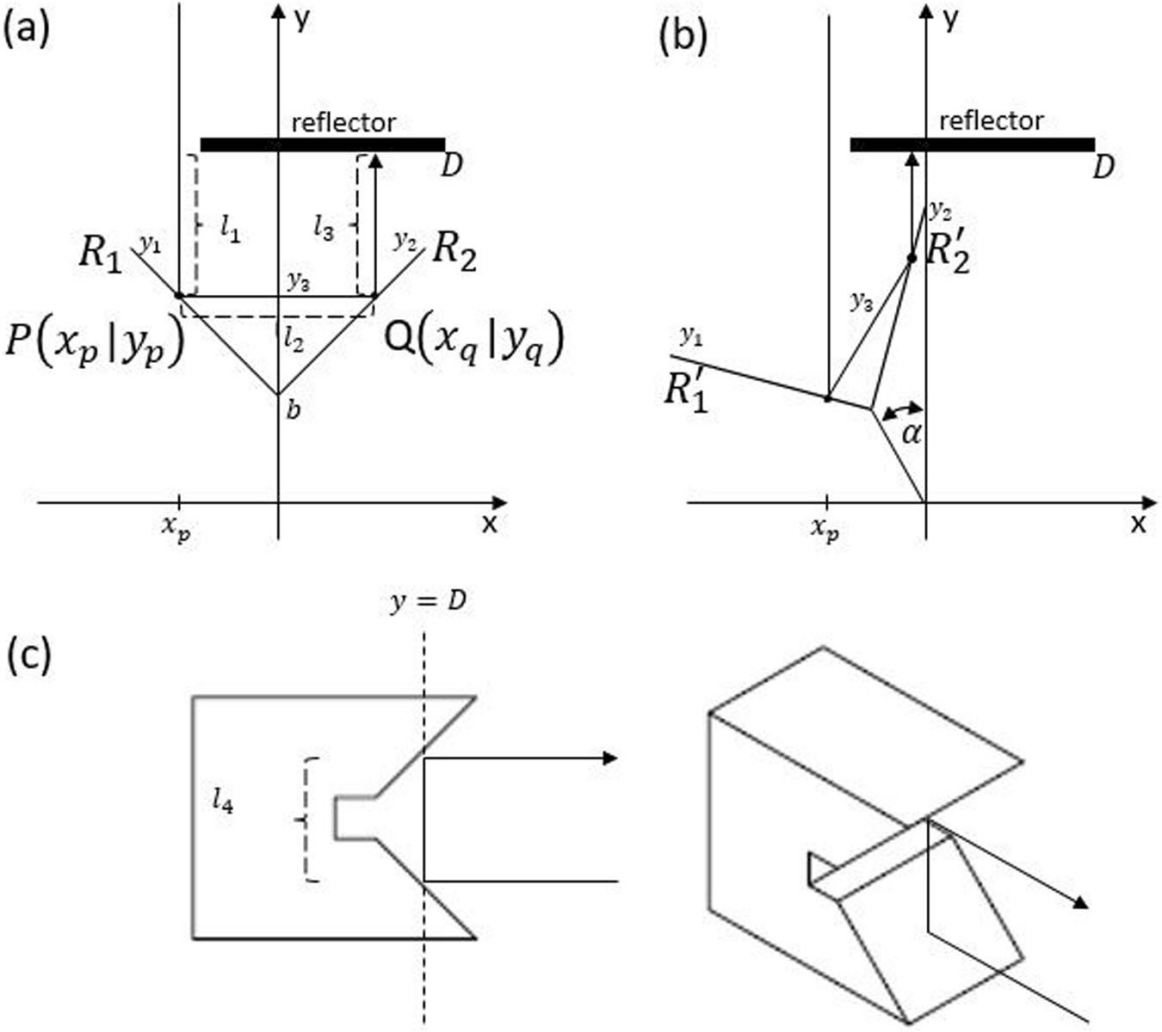
Schematic of the retro-reflector. **a** the delay line comprises a retro-reflector with reflective surfaces *R*_1_, *R*_2_, reflector at a distance D. The incoming and outgoing reflected beam paths are shown when entering the delay at *x* = *x*_*p*_. **b** Rotated retro-reflector by an angle *α* around the origin. **c** Schematic of a reflector placed at a distance D to elevate and to reflect back the beam.

The analytical expression for the angle-dependent optical path length within the rotational delay line can be found by computing the individual path lengths of the incoming and the reflected rays on the surfaces *R*_1_ and *R*_2_, and the reflector (Fig. 7a). To do so, we have first to compute how the reflective surfaces *R*_1_ and *R*_2_, described by the linear equations *y*_1_(*x*) and *y*_2_(*x*), are transformed under rotation around the origin. By rotating the reflective surfaces, which are described as:

$${R}_{1}:{y}_{1}= 	 -x+b\\ {R}_{2}:{y}_{2}= 	 \,x+b$$

by angle *α* around the *z*-axis (Fig. 7b) with the rotation matrix *M* = $$\left(\begin{array}{ll}\cos (\alpha )&-\sin (\alpha )\\ \sin (\alpha )&\cos (\alpha )\end{array}\right)$$ results in the following transformation:

10 $${R}_{1}^{{\prime} }:\left(\begin{array}{l}{x}^{{\prime} }\\ {y}_{1}^{{\prime} }\end{array}\right)=M\left(\begin{array}{l}x\\ {y}_{1}\\ \end{array}\right)$$

and

11 $${R}_{2}^{{\prime} }:\left(\begin{array}{l}{x}^{{\prime} }\\ {y}_{2}^{{\prime} }\end{array}\right)=M\left(\begin{array}{l}x\\ {y}_{2}\end{array}\right)$$

In the case of ${R}_{1}^{{\prime} }$, this can be simplified to:

$${x}^{{\prime} }=	\,(\cos (\alpha )+\sin (\alpha ))x-\sin (\alpha )b\\ {y}_{1}^{{\prime} }=	\,(\sin (\alpha )-\cos (\alpha ))x+\cos (\alpha )b$$

and expressing *x* in terms of ${x}^{{\prime} }$ and inserting into ${y}_{1}^{{\prime} }$ results in a general expression of a transformed *R*_1_ by angle *α*:

$${y}_{1}^{{\prime} }=\frac{sin(\alpha )-cos(\alpha )}{cos(\alpha )+sin(\alpha )}{x}^{{\prime} }+\frac{sin(\alpha )-cos(\alpha )}{cos(\alpha )+sin(\alpha )}sin(\alpha )b+cos(\alpha )b$$

Making use of trigonometric identity ($cos(\theta )+sin(\theta )=\sqrt{2}cos(\theta -\pi /4)$) and $sin(\theta )-cos(\theta )=\sqrt{2}sin(\theta -\pi /4)$ the above equation can be simplified to:

$${y}_{1}={S}_{1}x+{C}_{1}$$

with *S*_1_ = *tan*(*α* − *π*/4) and *C*_1_ = *cos*(*α*)*b* + *S*_1_*sin*(*α*)*b*. In analogy to the above derivation of ${y}_{1}^{{\prime} }$ this results for ${y}_{2}^{{\prime} }$ in:

$${y}_{2}^{{\prime} }=\frac{sin(\alpha )+cos(\alpha )}{cos(\alpha )-sin(\alpha )}{x}^{{\prime} }+\frac{sin(\alpha )+cos(\alpha )}{cos(\alpha )-sin(\alpha )}sin(\alpha )b+cos(\alpha )b$$

and with the help of trigonometric identity into:

$${y}_{2}={S}_{2}x+C2$$

with *S*_2_ = *tan*(*α* + *π*/4) and *C*_2_ = *cos*(*α*)*b* + *S*_2_*sin*(*α*)*b*. As shown in Fig. 7 the incoming optical beam enters into the optical rotational delay line and is reflected at the point *P*(*x*_*p*_∣*y*_*p*_) towards the reflective surface *R*_2_. In the following we will discuss the derivation of the angle-dependent reflected beam from surface *R*_1_ towards surface *R*_2_. Since the incoming optical beam enters at *x* = *x*_*p*_ we can assume that it is emitted from point *A*(*x*_*p*_∣*D*). The reflected beam can be constructed by mirroring the point *A* at the surface *R*_1_ and constructing a straight line through the point *P* and ${A}^{{\prime} }$. Let the mirror point of *A* have the following coordinates ${A}^{{\prime} }(h| k)$, the slope of the straight line through *A* and ${A}^{{\prime} }$ is given by ${S}_{A{A}^{{\prime} }}=\frac{D-k}{{x}_{p}-h}$. Since the reflective surface *R*_1_ is perpendicular to the straight line going through *A* and ${A}^{{\prime} }$, the product of these slopes equal to minus one $({S}_{A{A}^{{\prime} }}\cdot {S}_{1}=-1)$. From this equation we can derive equation for the x-coordinate of the point ${A}^{{\prime} }$:

12 $$h={S}_{1}(D-k)+{x}_{p}.$$

Moreover, we know that the middle point ${M}_{A{A}^{{\prime} }}(\frac{h+{x}_{p}}{2}| \frac{k+D}{2})$ between *A* and ${A}^{{\prime} }$ lies on the reflective surface *R*_1_. Inserting ${M}_{A{A}^{{\prime} }}$ into *y*_1_ results in:

13 $$k=\frac{{S}_{1}^{2}D+2{S}_{1}{x}_{p}+2{C}_{1}-D}{1+{S}_{1}^{2}}$$

The both derived expressions for *h* and *k* can now be used to compute the slope in the reflected beam in point P. The slope ${S}_{{A}^{{\prime} }P}$ is given by:

14 $${S}_{{A}^{{\prime} }P}=\frac{{y}_{1}({x}_{p})-k}{{x}_{p}-h}=\frac{{S}_{1}{x}_{p}+{C}_{1}-(\frac{{S}_{1}^{2}D+2{S}_{1}{x}_{p}+2{C}_{1}-D}{1+{S}_{1}^{2}})}{{x}_{p}-({S}_{1}(\frac{{S}_{1}^{2}D+2{S}_{1}{x}_{p}+2{C}_{1}-D}{1+{S}_{1}^{2}}-D)+{x}_{p})}=-\frac{1-{S}_{1}^{2}}{2{S}_{1}}$$

Since *S*_1_ = *tan*(*α* − *π*/4) the equation (3) can be further simplified with the double trigonometric angle identity *tan*(2*θ*) = 2*tan*(*θ*)/(1 − *tan*(*θ*)^2^) and *cot*(*θ*) = *tan*(*π*/2 − *θ*) to:

15 $${S}_{{A}^{{\prime} }P}=-\frac{1-{S}_{1}^{2}}{2{S}_{1}}=-\frac{1}{tan(2(\alpha -\pi /4))}=tan(2\alpha )$$

By knowing the slope ${S}_{{A}^{{\prime} }P}$ and the point *P*(*x*_*p*_∣*y*_1_(*x*_*p*_)) the reflected beam is described by the following equation:

16 $${y}_{3}={S}_{3}x+{C}_{3}$$

with *S*_3_ = *tan*(2*α*) and *C*_3_ = *C*_1_ + *S*_1_*x*_*p*_ − *S*_3_*x*_*p*_. Due to the law of reflection, the first reflected beam, going from point P to point Q and described by the linear equation *y*_3_(*x*), makes an angle 2*α* with the *x*-axis. Therefore, its slope is ${S}_{3}=\tan 2\alpha$. By knowing the value of *y*_3_(*x*_*p*_) we can solve for the offset *C*_3_:

17 $${y}_{3}({x}_{p})={S}_{3}{x}_{p}+{C}_{3}={y}_{1}({x}_{p})={S}_{1}{x}_{p}+{C}_{1}$$

18 $${C}_{3}={S}_{1}{x}_{p}-{S}_{3}{x}_{p}+{C}_{1}$$

The second point of the reflected beam in the rotation delay line is Q, whose x-coordinate can be found by setting *y*_3_(*x*) = *y*_2_(*x*). The results is *Q*(*T*∣*S*_3_*T* + *C*3) with $T=\frac{{C}_{2}-{C}_{3}}{{S}_{3}-{S}_{2}}$. With all the derived expressions we can compute the angle dependent path length *L*(*α*) inside the rotational delay line which consists of:

19 $$L=\mathop{\sum }\limits_{i=1}^{3}{l}_{i}(\alpha )+{l}_{4}$$

If the reflector is a plain mirror then *l*_4_ = 0 and in the case of a retro reflector as shown in Fig. 7c*l*_4_ is a constant which is given by the geometry design of the reflector. The lengths *l*_*i*_ are computed from the geometry as following:

20 $${l}_{1}=D-{y}_{1}({x}_{p})=D-{S}_{1}{x}_{p}-C1$$

21 $${l}_{2}=\sqrt{{(T-{x}_{p})}^{2}+{({S}_{3}T+{C}_{3}-{S}_{1}{x}_{p}-{C}_{1})}^{2}}$$

22 $${l}_{3}=D-{y}_{2}({x}_{q})=D-{S}_{3}T-{C}_{3}$$

23 $$L=D-{S}_{1}{x}_{p}-{C}_{1}+\sqrt{{(T-{x}_{p})}^{2}+{({S}_{3}T+{C}_{3}-{S}_{1}{x}_{p}-{C}_{1})}^{2}}+D-{S}_{3}T-{C}_{3}+{l}_{4}$$

### Alignment requirements for the rotational delay line

The only requirement for the system is a collimated beam over 1m (system dependent) with the beam diameter below 7 mm. The collimated beam is only required within the rotational delay line. Such conditions are relatively easy to achieve at the wavelength of 8 um and becomes easier with shorter wavelength. However, the beam diameter of the system can be increased by designing a rotating retro-reflector with a different height. The alignment of the system is relatively simple, since the entire alignment principle is based on retro-reflectors, which either reflects the beam back by shifting it in the plain (rotating retro-reflector) or lifting the beam in height and reflecting it back (static retro-reflector). We would like also to note that the polarisation of the system in preserved.

### Supplementary information

Scalari_Peer Review File

Supplementary Information

## Data Availability

The measurement data that support the plots within this paper are available from the corresponding author upon reasonable request. Data used to produce the figures of this Article is available in the ETH Research Collection^50^.
