# Supplementary material for: Frequency chirped Fourier-Transform spectroscopy
Source: Commun Phys. 2023 Mar 23;6(1):53. doi: 10.1038/s42005-023-01157-5 (PMC11041810; doi:10.1038/s42005-023-01157-5)
Supplement: Supplementary file 2 — Supplementary Information [file 42005_2023_1157_MOESM2_ESM.pdf]

Supplementary material for Frequency chirped Fourier-Transform spectroscopy

Sergej Markmann[1], Martin Frankié[1], Mathieu Bertrand[1], Mehran Shahmohammadi[1], Andres Forrer[1], Pierre Jouy[1], Mattias Beck[1], Jérôme Faist[1] and Giacomo Scalari[1]

[1]Institute for Quantum Electronics, ETH Zürich, 8093 Zürich, Switzerland

1 Surface quality of the rotational delay line

The figure S1 below shows a standard deviation of the mean of four beams, two static beams and two Doppler shifted beams an the sample and normalization detector. The Doppler/static beams were recorded by blocking the static/Doppler beams.

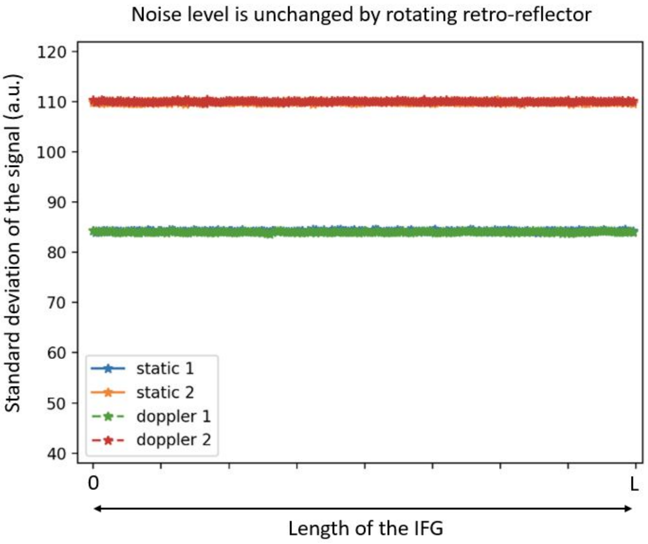

Figure S1: **surface roughness of the rotational delay line**

The standard deviation of static 1 and Doppler 2 beam are constant and do not change as a function of time and hence rotation angle. The same is valid for static 2 and Doppler 1 beams. This directly shows that STD is the

same for Doppler shifted and static beam. The difference in the STD of the beam is due to the power difference of the beams on the detector. Hence, we can be sure that surface roughness quality (< 10nm Ra) achieved with a diamond turning is sufficient for our applications.

2 Mid-IR detector response curve

In Fig. S2 we display the measured response of the detectors and the employed beam power.

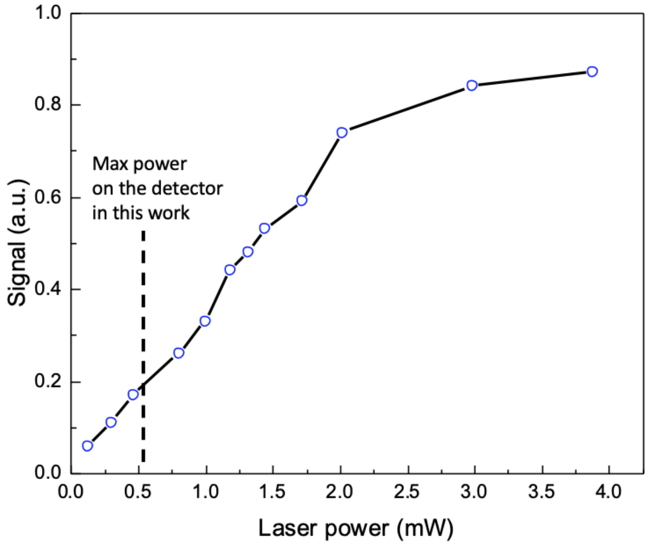

Figure S2: **Response of the employed Mid-IR detectors**. Dashed line indicates the operating power of the spectrometer's laser beams

3 Visibility of the system

The visibility is constant over the total path length and is larger than 85%. The figure S3 shows the non-resampled interferogram (IFG) of the QCL in the single mode regime, just above the laser threshold with a total DC power of 40uW on the detector.

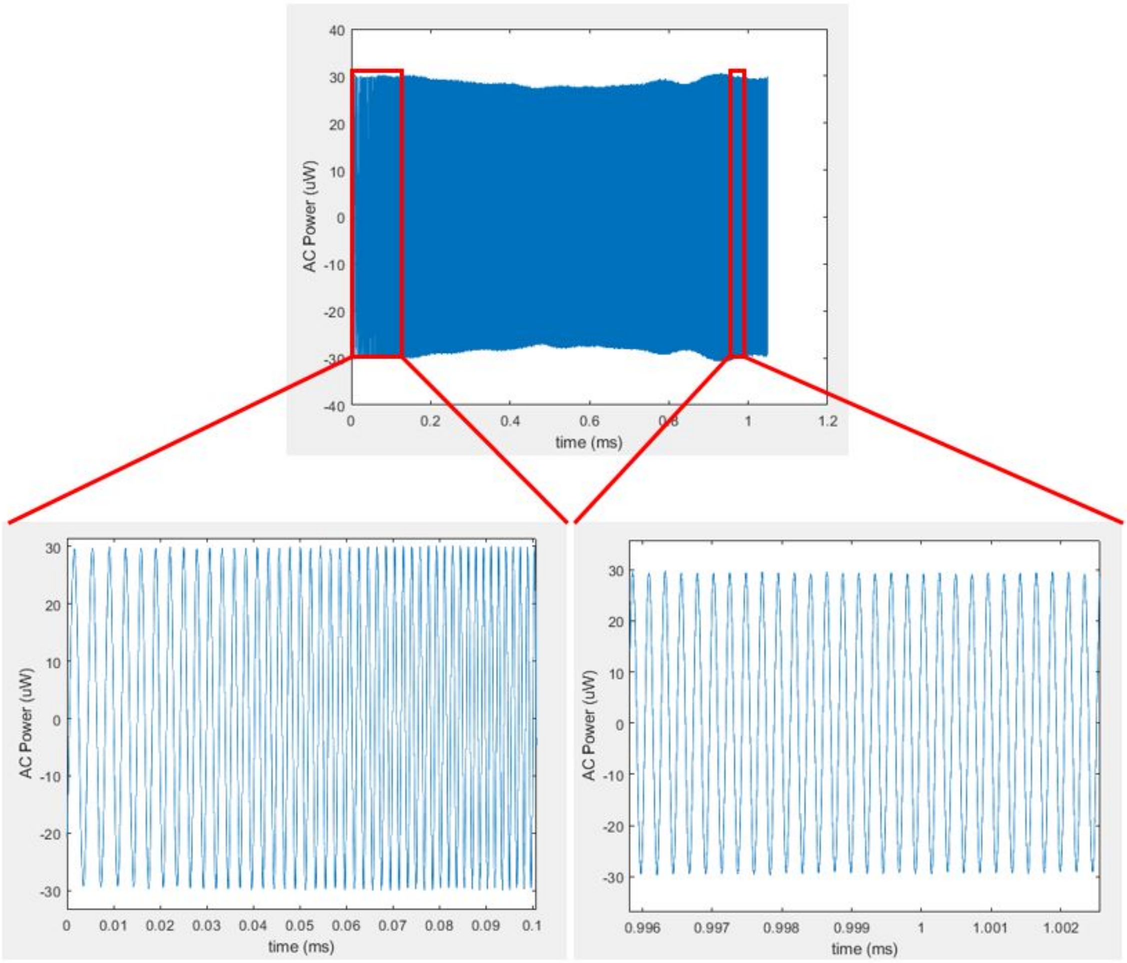

Figure S3: **Interferogram visibility.** Single mode QCL interferogram at the beginning and at the end of the delay scan.
