# Supplementary material for: Frequency chirped Fourier-Transform spectroscopy
Source: Commun Phys. 2023 Mar 23;6(1):53. doi: 10.1038/s42005-023-01157-5 (PMC11041810; doi:10.1038/s42005-023-01157-5)
Supplement: Supplementary file 1 — Scalari_Peer Review File [file 42005_2023_1157_MOESM1_ESM.pdf]

nature portfolio

Peer Review File

Frequency chirped Fourier-Transform spectroscopy

Open Access This file is licensed under a Creative Commons Attribution 4.0 International License, which permits use, sharing, adaptation, distribution and reproduction in any medium or format, as long as you give appropriate credit to the original author(s) and the source, provide a link to the Creative Commons license, and indicate if changes were made. In the cases where the authors are anonymous, such as is the case for the reports of anonymous peer reviewers, author attribution should be to 'Anonymous Referee' followed by a clear attribution to the source work. The images or other third party material in this file are included in the article's Creative Commons license, unless indicated otherwise in a credit line to the material. If material is not included in the article's Creative Commons license and your intended use is not permitted by statutory regulation or exceeds the permitted use, you will need to obtain permission directly from the copyright holder. To view a copy of this license, visit http://creativecommons.org/licenses/by/4.0/.

Reviewers' comments:

Reviewer #1 (Remarks to the Author):

The manuscript entitled "Frequency chirped Fourier-Transform spectroscopy" demonstrates rapid-scan Fourier-transform spectroscopy with a quantum cascade laser. The authors developed a delay scanner based on a rotating retro-reflector with multiple facets and demonstrated high-resolution spectroscopy with a resolution of 0.5 cm-1 at a scan rate of about a kHz. The authors used the system and demonstrated spectrally interleaved high-resolution spectroscopy with the help of the frequency comb nature of the light source.

The manuscript is overall well written, and the experimental results are shown at the level of journal publication. However, the demonstrated technique has no big conceptual significance over the existing systems. As mentioned in the manuscript, the concept of high-speed Fourier-transform spectroscopy with a rapid delay scanner with rotational motion rather than a linear one has already been demonstrated in various methods (some of them showed even faster scanning), and the comb-interleaved high-resolution Fourier-transform spectroscopy has also been demonstrated. Therefore, I only see the significance of the current manuscript in the combination of a rapid-scan interferometer and a quantum cascade laser comb, which is a rather incremental step in the field.

Comments

1. In the introduction, it is written as "Recently, successful proof-of-concept of phase-controlled FT spectroscopy [13] and time-stretch spectroscopy [14–16] have been demonstrated, which …. However, these newly developed methods rely on dispersive elements such as gratings, dispersive fibers, or prisms. Hence, they can be operated only over a limited optical bandwidth, mainly at visible and near-infrared frequencies." This statement does not correctly refer to the previous works. It is true that the refs [13-16] use dispersive optical elements such as gratings or optical fibers, but why does it limit the operational spectral band only in the visible and near-infrared? Optical gratings also work broadband in the mid-infrared, including the fingerprint region. For example, the phase-controlled FT spectroscopy [13] is also demonstrated in the mid-infrared region [Hashimoto et al., Laser&Photonics Reviews 15, 2000374 (2021)], which the authors should refer to. Ref [14] is also operated in the mid-infrared region with an optical grating. I would suggest the authors appropriately modify the statement.

2. On page 3, there is a statement, "By taking the advantages and disadvantages of the above described optical delay systems into account, we can define criteria which the desired system should fulfill: (I) Support of a large optical bandwidth; hence dispersive elements have to be avoided." Similar to comment 1, why do the dispersive elements have to be avoided for a large optical bandwidth? The dispersive elements do not support infinitely large bandwidth but surely work at "broadband." The authors should discuss more in detail quantitatively (e.g., how large bandwidth, etc.) if keeping this statement.

3. I do not understand the sentence, "The use of a rotational element decouples the spectral resolution from the interferogram acquisition time." It sounds strange saying that the spectral resolution is decoupled from the interferogram acquisition time. The spectral resolution and measurement time must always be coupled (Fourier-transform relation) in any FT techniques. A different thing from the conventional system is just the scan speed, which is not related to the time-frequency coupling. I would suggest the authors reconsider the statement.

4. In section 2.4, there is a statement, "Thus, with the employed detectors and the laser, the SNR can be improved by longer integration times or by more powerful laser, since the detector saturation power is around 8 mW, …." The detector saturation power (8 mW) sounds very high compared to conventional ones in the mid-infrared region. I would imagine the saturation power of a typical mid-infrared detector is lower by an order of magnitude. Does the detector linearly respond up to such a

high average power? I would request the authors to provide the data showing the linearity.

5. As the authors mention in the manuscript, the flatness and angle of the 8 RDL facets are critical in FT spectroscopy performance. Fig 2(d) shows clear discrepancies in each interferogram. Since it is the heart of the technique, I suggest the authors make more effort to evaluate them. For example, the 8 interferograms could be displayed in a larger manner so that one can see the difference between them. It would also be helpful to see the residuals of the difference. If SNR is not enough with a single interferogram, one could average interferograms measured with each facet and show the comparison.

6. How good is the visibility of the interferometer? Is it constant over the entire delay scan? I would request the authors to discuss it.

7. The absorption lines of the measured CH4 spectrum (Fig.5) have broader width than the HITRAN spectrum. What causes the broadening?

8. The parameters of the theoretical etalon spectrum in Fig.5 are missing. Is it calculated with experimentally measured etalon's properties or a fitted curve? I would request to make it clear.

9. In this manuscript, the authors state that the combination of an interferometer and a QCL source performs dual-comb spectroscopy with a single source. In my opinion, one should not call such a system "dual-comb." Otherwise, all the FT spectroscopy systems which use a coherent laser and an interferometer are categorized in the dual-comb spectroscopy, which is too broad. I would suggest calling it "comb spectroscopy" if one wants to emphasize the nature of the frequency comb.

Reviewer #2 (Remarks to the Author):

The paper by S. Markmann, et al., demonstrated a fast Mid-IR Fourier transform spectrometer by using an optical frequency comb (OFC) as light source and by implementing a rotational delay line (RDL) to improve acquisition speed. The mid-IR QCL allows molecular spectroscopy at 8 micrometer mid-IR band. The rotational delay line effectively decouples the spectral resolution from the interferogram acquisition time. This setup allows dual-comb spectroscopy by taking advantage of the Doppler-shifting of the comb in the RDL. The transmission resonances of a silicon etalon and the absorption lines of a Doppler-broadened low-pressure methane sample (CH4) have been characterized with a < 250 MHz resolution. The time-resolved high spectral resolution spectroscopy has also been demonstrated. Overall, this manuscript is well organized and written. The RDL-based scheme provides an alternative strategy to achieve dual-comb spectroscopy with a single comb, while the sophisticated dual-comb phase-locking apparatus are avoided. This work may draw broad interest in the field of molecular spectroscopy and optical frequency comb applications, in my opinion. Therefore, I recommend to publish this work in Commun. Phys. Minor revision is required, as follows,

1. What's the frequency stability of the Doppler shifted OFC after RDL given the mechanical RDL may show non-uniform rotation? Have the authors ever monitored the long-term and short-term frequency stability of differential repetition rate frequency (delta_f_frep) which is frequently used as an indicator for dual-comb stability?
2. Whether the f_ceo of the QCL is stable and by what cavity parameter is this frequency determined? The impact of f_ceo on frequency chirped FTS performance may need to be discussed.
3. Related with Q2, is it possible to retrieve phase information from the FTS in the absence of f_ceo information?

Reviewer #3 (Remarks to the Author):

Markmann et al discusses temporal control of tetrahertz FT spectrometer using efficiency of reflective

specially shaped mirrors. This is a highly topical area of real time spectroscopy at near infrared wavelength. Due to the limitations in mirror designs, path length difference are limiting to less than few cm. The authors has presented a mirror scanning solution that can reach ~ 3 cm. The author has presented evidence that the scanning technology provides advantages in speed without reducing resolution (frequency scanning). Using the constructed new delay line, the authors applied to detect gas absorption at sub-GHz resolution. Overall, the reported technique is interesting and could be of interest for the Tetrahertz community that has often rely on lower efficiency and slow delay line. However, a major problem of the manuscript looks to be a chapter in a thesis that is not clearly written for a broader applied physics audience. For that, significant changes to the presentation as well more more focus on the technical novelty (not just measurement and calibration) is required before the manuscript can be considered for publication. A Major revision is required.

Major Points
- Central to the paper is design of the rotational delay line and a static retro-reflector. That is clearly a unique and clever optical design. The authors claimed that the surface roughness of less than 10 nm do not contribution to intensity noise, is unclear and do not appear to be well supported. It is necessary to consider roughness here with wavelength should be include in the supplementary information. From Fig.2, there is clear ripples from the rotational scanning (4), (8). It is unclear if those intensity ripples are created due to alignment of the static retroreflector? or the machining of the mirrors. This should be better discussed.
- The authors tried to provide information of rotating delay line and beam entrance. There is not discussion on divergence and beam waist over multiple reflectors. This is likely to be a fairly challenging steps that does not appear to be discussed clearly.
Alignment protocol for such a unique mirror scanning method would require significant alignment steps. For that, it will be greatly helpful for the authors to include so that readers are aware of the limitations here in beam waist and scanning angle. This is particularly important because clipping beam waist onto each scanning mirror will create poor reflectance.
- Section 3 of the paper talks about methane detection. While this is a good proof of concept, the discussion on the results do not clearly assert the sub-GHz resolution against current THz spectroscopy system. This lack of the discussion will proof difficult for readers to assess the advantage of such a rotational delay line.

Minor comments
- There are generally a lack of attention on how the novelty of the rotational delay line has been presented. While the author shows that such a delay line can be useful, it rather unclear how they have clearly convincingly shown to surpass speed current THz spectroscopy system for both resolution and speed. The author keep mentioning about state of the art. But it is unclear what that actually is?

- In general, the paragraph are lengthy and feel more like a quick submission from a cut in a thesis page. Sentences do not provide clear concise discussions necessary in a research article.

1) Long sentences can be cut down significantly for example
"Providing sub-GHz spectral resolution shows a clear advantage of our approach, since such a high resolution is challenging to achieve with the sate-of-the-art FT spectrometers which are typically on the order of 0.2 cm^{-1} (6 GHz) or 0.06 cm^{-1} (1.8 GHz)
for high resolution spectroscopy". Could be rephrase as

Our rotational delay line achieve resolution of on the order of 0.2 cm^{-1} (6 GHz) or 0.06 cm^{-1} (1.8 GHz) close to the state of the art FT spectrometers.

We would like to thank all the thee Reviewers for their interest in our manuscript and taking the time to carefully evaluate it. We believe the answers provided below to each point raised by the Reviewers will improve the manuscript significantly and warrant publication in Communications Physics. All changes in the main manuscript and in the supplementary material are marked with red color.

Answers to Reviewer 1

The manuscript entitled "Frequency chirped Fourier-Transform spectroscopy" demonstrates rapid-scan Fourier-transform spectroscopy with a quantum cascade laser. The authors developed a delay scanner based on a rotating retroreflector with multiple facets and demonstrated high-resolution spectroscopy with a resolution of 0.5 cm-1 at a scan rate of about a kHz. The authors used the system and demonstrated spectrally interleaved high-resolution spectroscopy with the help of the frequency comb nature of the light source.

The manuscript is overall well written, and the experimental results are shown at the level of journal publication. However, the demonstrated technique has no big conceptual significance over the existing systems. As mentioned in the manuscript, the concept of high-speed Fourier-transform spectroscopy with a rapid delay scanner with rotational motion rather than a linear one has already been demonstrated in various methods (some of them showed even faster scanning), and the comb-interleaved high-resolution Fourier-transform spectroscopy has also been demonstrated. Therefore, I only see the significance of the current manuscript in the combination of a rapid-scan interferometer and a quantum cascade laser comb, which is a rather incremental step in the field.

We are happy that the Reviewer finds our results well presented, and we thank her/him for the useful comments. It is clear that we did not present clearly enough the advantages of our results. However, we strongly disagree with the notion that our results are incremental and that similar systems have been demonstrated before. To our knowledge, there has never been any demonstration of a rotational Fourier-transform spectroscopy system without a dispersive element. We discussed this point in our Introduction:

Recently, proof-of-concept of phase-controlled FT spectroscopy [13]

and time-stretch spectroscopy [14–16] have been successfully demonstrated, which outperform the acquisition speed of traditional FT spectrometer by several orders of magnitude, while maintaining a relatively high spectral resolution. However, these newly developed methods rely on dispersive elements such as gratings, dispersive fibers, or prisms. Hence, they can be operated only over a limited optical bandwidth, mainly at visible and near-infrared frequencies.

. . .

Several optical delay line systems have been developed which are based on a rotational delay line, such as a circular involute stage [22] and curvilinear reflectors [23], [24]. These two systems have in common that the optical path difference is angle dependent, obtained via rotational optics which guide the incoming beam to a static reflector which self reflects the beam back. Hence, in such systems the incoming and out going beam are anti-parallel, which will result in large undesired feedback into the laser source. Moreover, these systems do not support extended light beams and hence pose a phase-dependent-delay across the beam diameter, which make them impractical for usage in the FT spectrometer. On the other hand, a compact flexible multi-pass rotary delay line using spinning micro-machined mirrors have been developed [25]. The big disadvantage of these systems is relatively small achievable optical path difference, which is typically less than 1 mm, and the requirement of fiber optics and optical circulators, thus limiting this system to a narrow optical bandwidth. Another interesting approach was shown in ref. [26] where a rotating retro-reflector in combination with a static plain mirror is used. The incoming beam which is guided via a rotational retro-reflector to the plane mirror is back reflected. The disadvantage of this system lies in a not separable incoming and outgoing beams. Moreover, the system size grows linearly with the increase of the optical achievable delay path. This make the system impractical for usage of desired delay lengths of 3 cm and more at large acquisition speeds.

For example, Ref. [13] uses a rotational mirror in conjunction with a dispersive grating, which results in a different Doppler shift for each frequency.

In contrast, our setup gives the same Doppler shift for each mode, and in addition separates the output from the input while avoiding any beam walks. This has not been done before, since it is necessary to compensate the divergence caused by the rotating mirror. It is true that the system in [13] is much faster than our demonstration. However, this is due to the use of a polygonal mirror which at the same time severely limits the spectral resolution to the degree where the frequency comb modes cannot be resolved. Finally, our proof-of-concept system could be made at least 10 times faster by using a faster motor.

Regarding the interleaving technique, we do not claim to propose it for the first time here. Rather, we show that in our system it is possible to implement it by the use of a single frequency comb source. Interleaving has been mostly realized with dual-comb systems, again suffering from the disadvantages discussed in the main text. It has also been done with FTIRs in slow scan mode, which is orders of magnitude slower, e. g. in [Piotr Maslowski et al., PHYSICAL REVIEW A 93, 021802(R) (2016)], which we have added as a new reference. In contrast, this is impossible with a rapid-scan FTIR since it cannot resolve the modes of a QCL comb. Here, our system is different than any other we know of, since it allows to do interleaving at speeds greatly surpassing rapid-scan FTIRs with a single comb source.

Even without considering the QCL comb as a unique source, our setup is unlike any other known to us, as it allows for very broad band operation (from THz to optical) and is faster than any rapid scan FTIR due to the physical limitations of a linearly accelerated stage. Thus, also the RDL (rotational delay line) in itself constitutes a significant advance in the field.

1. In the introduction, it is written as "Recently, successful proof-of-concept of phase-controlled FT spectroscopy [13] and time-stretch spectroscopy [14–16] have been demonstrated, which . . . . However, these newly developed methods rely on dispersive elements such as gratings, dispersive fibers, or prisms. Hence, they can be operated only over a limited optical bandwidth, mainly at visible and near-infrared frequencies." This statement does not correctly refer to the previous works. It is true that the refs [13-16] use dispersive optical elements such as gratings or optical fibers, but why does it limit the operational spectral band only in the visible and near-infrared? Optical gratings also work broadband in the mid-infrared, including the fingerprint region.

For example, the phase-controlled FT spectroscopy [13] is also demonstrated in the mid-infrared region [Hashimoto et al., Laser and Photonics Reviews 15, 2000374 (2021)], which the authors should refer to. Ref [14] is also operated in the mid-infrared region with an optical grating. I would suggest the authors appropriately modify the statement.

We agree with the Reviewer that the use of dispersive elements can also be relatively broadband, also covering the fingerprint region from 8-12 μm (see e. g. [Wang et al., Physics Letters A, 381, pp. 1439-1444 (2017)]. The grating in [Hashimoto et al., Laser and Photonics Reviews 15, 2000374 (2021)] to which the reviewr refers reports a maximum bandwidth of their system limited to 300 cm^{-1}. We would like to clarify the statement mentioned above, as we recognize that it could be misinterpreted. Gratings of limited bandwidth are of course available across a wide spectral region. However, a continuous coverage from optical frequencies down to THz is challenging to achieve with a single dispersive element as used in Ref [13-16] and the two references mentioned above. In our approach, only reflective elements are used, covering continuously the frequency band from optical (even UV) down to THz frequencies with almost always above 90% reflectivity. We are not aware of any grating that comes close to these specifications. We have modified the statement in the manuscript to make this point more clear.

2. On page 3, there is a statement, "By taking the advantages and disadvantages of the above described optical delay systems into account, we can define criteria which the desired system should fulfill: (I) Support of a large optical bandwidth; hence dispersive elements have to be avoided." Similar to comment 1, why do the dispersive elements have to be avoided for a large optical bandwidth? The dispersive elements do not support infinitely large bandwidth but surely work at "broadband." The authors should discuss more in detail quantitatively (e.g., how large bandwidth, etc.) if keeping this statement.

By discussing about the broad band range we imply a bandwidth typical for an FTIR from 1 micrometer (299.8 THz) to 20 micrometers (14.9 THz). We are not aware of any dispersive element which could cover continuously the above mentioned bandwidth. We have now stated the discussed bandwidth in the manuscript.

3. I do not understand the sentence, "The use of a rotational element de-

couples the spectral resolution from the interferogram acquisition time.” It sounds strange saying that the spectral resolution is decoupled from the interferogram acquisition time. The spectral resolution and measurement time must always be coupled (Fourier-transform relation) in any FT techniques. A different thing from the conventional system is just the scan speed, which is not related to the time-frequency coupling. I would suggest the authors reconsider the statement.

We agree with the Review that we need to discuss this statement. The spectral resolution in our system is constant and is given by the total optical path length of the rotational delay line. The acquisition time for the IFG can be adjusted and is only limited by the motor angular velocity. This is different to the state-of-the-art FTIR with the linear delay line. To clarify this point even further, the “measurement time” of a spectrum is inversely proportional to the “scan speed”, agreed by the Reviewer to be de-coupled from the resolution. We believe this misunderstanding stems from the fact that in our system, the measurement time is coupled to the beating frequency between the Doppler-shifted and static beams, which is different from the resampled frequency, corresponding to the actual frequencies of the source. We would therefore like to keep the present formulation in the manuscript:

> The use of a rotational element decouples the spectral resolution from the interferogram acquisition time. This is due to the fact, that the spectral resolution is fixed and is given by the total optical path length of the rotational delay line. The temporal resolution is given by the angular velocity of the rotational optical element and is independent on the total optical path length.

4. In section 2.4, there is a statement, “Thus, with the employed detectors and the laser, the SNR can be improved by longer integration times or by more powerful laser, since the detector saturation power is around 8 mW, ...” The detector saturation power (8 mW) sounds very high compared to conventional ones in the mid-infrared region. I would imagine the saturation power of a typical mid-infrared detector is lower by an order of magnitude. Does the detector linearly respond up to such a high average power? I would request the authors to provide the data showing the linearity.

We use a detector module: PVMI-4TE-10.6+MIP-10k-100M from Vigo systems. According to the company characterisation this detector saturates

around 8 mW. The saturation level of these detectors is mainly limited by the pre-amplifier (bandwidth) and the area of the detector. Saturation of hundreds of uW, to which the Reviewer is referring, are typical for MCT detectors with the detector areas of 50 um x 50 um. The power on the detector in our experiment is in the order of 500 uW, with a detector area of 1 mm x 1 mm which clearly is in the linear regime even for small-area detectors. Any saturation effect would appear in a non symmetric or clipped signal which we do not observe. For any further information about the saturation power of our detector the Reviewer and readers of the manuscript can contact Vigo systems directly, as we have already specified which detector we used in the manuscript (detector PVMI-4TE-10.6+MIP-10k-100M).

5. As the authors mention in the manuscript, the flatness and angle of the 8 RDL facets are critical in FT spectroscopy performance. Fig 2(d) shows clear discrepancies in each interferogram. Since it is the heart of the technique, I suggest the authors make more effort to evaluate them. For example, the 8 interferograms could be displayed in a larger manner so that one can see the difference between them. It would also be helpful to see the residuals of the difference. If SNR is not enough with a single interferogram, one could average interferograms measured with each facet and show the comparison.

As we have mentioned, fabrication of the rotation element is crucial and need to be done with care. We took special care with respect to surface quality of all static and rotating retro reflectors by surface polishing via diamond turning. This ensured a surface roughness $<$ 10 nm Ra. Fig 2(d) shows IFGs recorded on each of the retro reflectors to show the operation principle. We would like to clarify that all measurements shown in from Fig.3 to Fig.6 have been realised on a single and always the same retro reflector, which enormously simplify the data acquisition and data treatment. Co-adding the interferograms from different retro reflectors as suggested by the Reviewer, is not the best strategy in our opinion. Rather, in order to take slight fabrication imperfections into account, one would record one sample transmission T (see main manuscript) for each retro reflector. The transmission T from each retro reflector can then be averaged. While such a data treatment would improve the SNR of the spectrometer by a factor $\lesssim 3$, it would not alter the main results and conclusions, and we therefore considered it beyond the scope of this proof-of-principle demonstration.

6. How good is the visibility of the interferometer? Is it constant over the entire delay scan? I would request the authors to discuss it. Yes, the visibility is constant over the total path length and is larger than 85%. The figure below shows the non-resampled IFG of the QCL in the single mode regime, just above the laser threshold with a total DC power of 40uW on the detector.

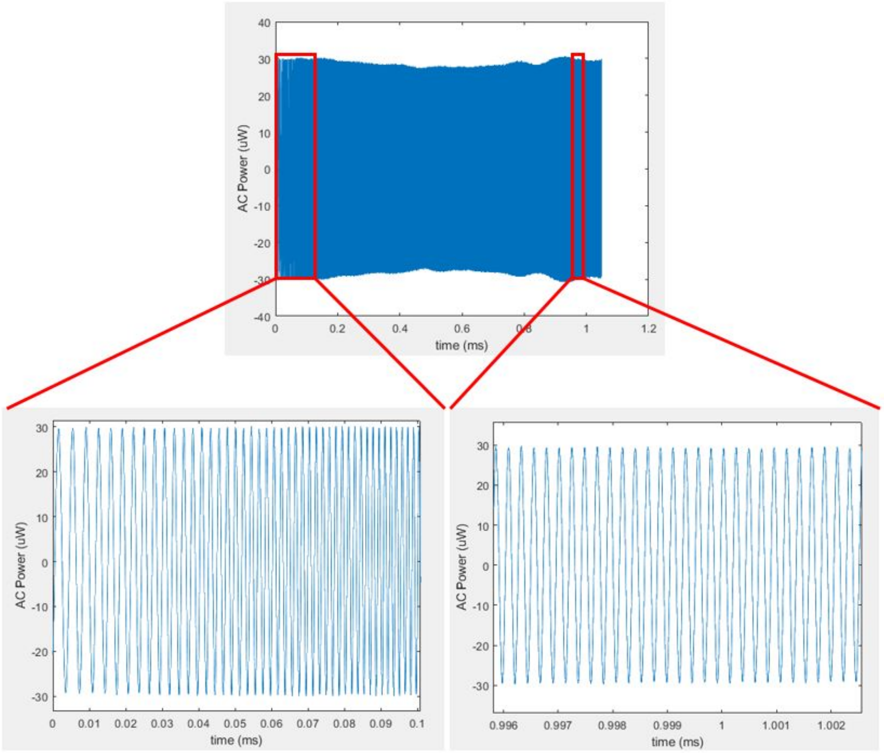

Figure 1: **single mode IFG**

7. The absorption lines of the measured CH4 spectrum (Fig.5) have broader width than the HITRAN spectrum. What causes the broadening?

The broadening of some absorption lines with respect to the HITRAN database can have several origins. Spectral leakage occurs if the source laser is not a perfect comb over its whole bandwidth. We can notice this effect as a smooth roll-off on each side of the spectrum in Fig. 4(a). During interleaving a current ramp is applied to the laser, which reduces laser stability and hence

increases spectral leakage. Consequently, in the situation where a neighboring line tunes into an absorption line, the intensity of a specific line will also be modulated. This could explain the broad features observed on the 2nd and 3rd lines from the right in Fig. 4(a), as well as the narrow features of higher than unity transmission throughout the spectrum, occurring at multiples of one f_{rep} from the methane absorption lines. Finally, the pressure gauge has an uncertainty of 10 mbar, which could explain a small part of the difference in linewidth. We have added this discussion to the main manuscript.

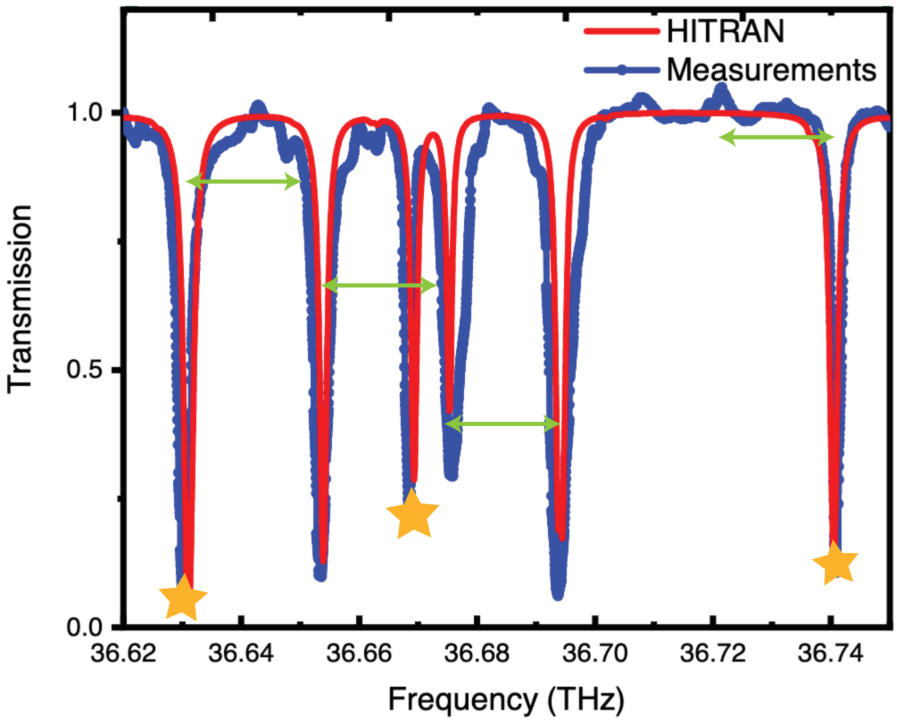

Figure 2: **Zoom into CH4 high resolution spectroscopy. Lines marked with stars have a correct line shape.**

8. The parameters of the theoretical etalon spectrum in Fig.5 are missing. Is it calculated with experimentally measured etalon's properties or a fitted curve? I would request to make it clear.

We thank the Reviewer for pointing it out. In Fig. 5 (a) and (d) we show the measurements results (black dots) and the computed theoretical etalon (red curve), so the theoretical etalon is provided. We made it more clear in

the figure caption.

9. In this manuscript, the authors state that the combination of an interferometer and a QCL source performs dual-comb spectroscopy with a single source. In my opinion, one should not call such a system "dual-comb." Otherwise, all the FT spectroscopy systems which use a coherent laser and an interferometer are categorized in the dual-comb spectroscopy, which is too broad. I would suggest calling it "comb spectroscopy" if one wants to emphasize the nature of the frequency comb.

We disagree in this aspect with the Reviewer, since we record a heterodyne signal originating from the beating of a Doppler-shifted and a non Doppler-shifted frequency comb. The maximum RF beating frequency of an 8 um laser is at 8 MHz which is limited by the speed of our motor. Conventional FTIR record a a signal around DC and the rapid scan FTIR do not have the resolution (typical 24 cm-1 or 36 cm-1) to resolve the comb lines (0.5cm-1). With this regard we would like to keep the terminology dual-comb spectroscopy.

Answers to Reviewer 2

The paper by S. Markmann, et al., demonstrated a fast Mid-IR Fourier transform spectrometer by using an optical frequency comb (OFC) as light source and by implementing a rotational delay line (RDL) to improve acquisition speed. The mid-IR QCL allows molecular spectroscopy at 8 micrometer mid-IR band. The rotational delay line effectively decouples the spectral resolution from the interferogram acquisition time. This setup allows dual-comb spectroscopy by taking advantage of the Doppler-shifting of the comb in the RDL. The transmission resonances of a silicon etalon and the absorption lines of a Doppler-broadened low-pressure methane sample (CH4) have been characterized with a ¡ 250 MHz resolution. The time-resolved high spectral resolution spectroscopy has also been demonstrated. Overall, this manuscript is well organized and written. The RDL-based scheme provides an alternative strategy to achieve dual-comb spectroscopy with a single comb, while the sophisticated dual-comb phase-locking apparatus are avoided. This work may draw broad interest in the field of molecular spectroscopy and optical frequency comb applications, in my opinion. Therefore, I recommend to publish this work in Commun. Phys. Minor revision is required, as follows,

We thank the Reviewer for the very positive assessment of our work.

1. What's the frequency stability of the Doppler shifted OFC after RDL given the mechanical RDL may show non-uniform rotation? Have the authors ever monitored the long-term and short-term frequency stability of differential repetition rate frequency (Δf_{rep}) which is frequently used as an indicator for dual-comb stability?

In contrast to the traditional dual-comb spectroscopy where Δf_{rep} is measured, we measure directly the f_{rep} of the laser which we down mix electrically with the help of an LO. Since we record a single IFG within one ms the resolution of our f_{rep} is 1kHz. We do not observe fluctuations larger than 1 kHz. The long term stability of the QCL beat note itself was measured (see figure below).

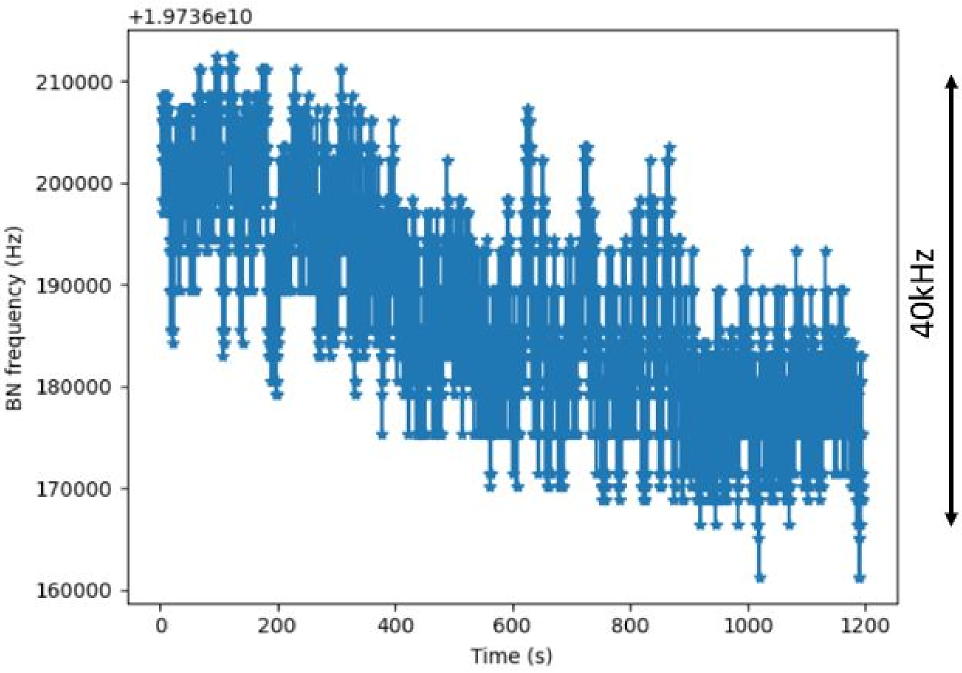

Figure 3: **Long term frequency stability of the** f_{rep}

We do agree with the Reviewer that non-uniform rotation may be present

due to mechanical instability of the rotation delay line. Since the reference laser and the mid-IR laser are co-linear and experience the same delay any possible non-uniform rotation will be imprinted also on the reference laser. Since the reference laser is used to resample the IFG any non-uniform rotation will be ideally removed from the mid-IR IFG. However, we also have studied the non-uniform motor rotation. The figure below shows a spectrum of the recorded mean value of the detector when one of the beams are blocked (static or Doppler beam).

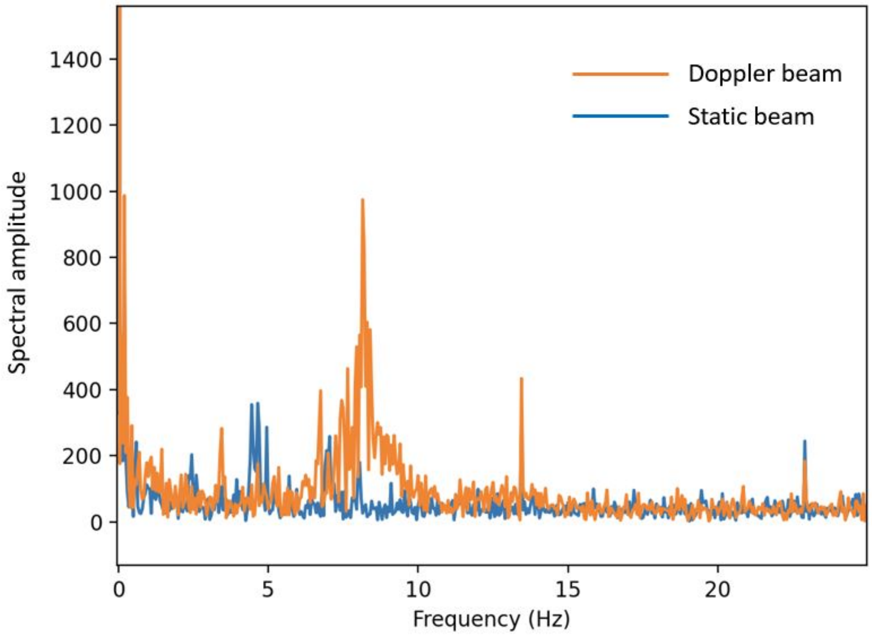

Figure 4: **mechanical stability of the RDL**

We do observe that the mean value of the static beam has peaks around 5 Hz and the Doppler beam around 8 Hz. These peaks in the spectrum of the static beam we attribute to some mechanical vibrations in the system. The peak of a Doppler beam at 8 Hz could point to a possible precession of a rotating retro-reflector or slightly different mechanical resonance. We would like to point out that the rotating retro-reflector was not balanced, so in the case of precession, the resonance at 8 Hz could be eliminated by balancing. A precession could lead to the change in the amplitude of the signal. However, the amplitude stability was study (see Fig.4 in the main text).

2. Whether the f_{ceo} of the QCL is stable and by what cavity parameter is this frequency determined? The impact of f_{ceo} on frequency chirped FTS performance may need to be discussed.

Since we measure directly the f_{rep}, we also use this information to compute f_{ceo} from the carrier wave. Before cutting the IFG to the length of $1/f_{rep}$ the chirped IFG is resampled. The resampling effectively removes a chirp induced by the non-linear optical path delay, because the reference CW laser (1550nm) experience the same frequency chirp. The f_{ceo} is then computed by evaluating a phase difference of the carrier wave at the beginning and the end $1/f_{rep}$ of the IFG. Since the IFG is resampled, there is no impact on f_{ceo} from the frequency chirp. Any variation of the f_{ceo} during the IFG acquisition can not be determined. The stability of the f_{ceo} is manly determine by the temperature stability control (in our case 10 mK) and any possible feedback which we try to minimize by employing optical isolator with 30dB isolation ratio.

3. Related with Q2, is it possible to retrieve phase information from the FTS in the absence of f_{ceo} information?

It is not possible to retrieve phase information.

Answers to Reviewer 3

Markmann et al discusses temporal control of tetrahertz FT spectrometer using efficiency of reflective specially shaped mirrors. This is a highly topical area of real time spectroscopy at near infrared wavelength. Due to the limitations in mirror designs, path length difference are limiting to less than few cm. The authors has presented a mirror scanning solution that can reach 3 cm. The author has presented evidence that the scanning technology provides advantages in speed without reducing resolution (frequency scanning). Using the constructed new delay line, the authors applied to detect gas absorption at sub-GHz resolution. Overall, the reported technique is interesting and could be of interest for the Tetrahertz community that has often rely on lower efficiency and slow delay line. However, a major problem of the manuscript looks to be a chapter in a thesis that is not clearly written for a broader applied physics audience. For that, significant changes

to the presentation as well more more focus on the technical novelty (not just measurement and calibration) is required before the manuscript can be considered for publication. A Major revision is required.

We would like to point out that the demonstration of proof-of-concept is done in the mid-IR range and not at THz (<10 THz) frequencies . We agree with the Reviewer 3 that this technology is also of high interest in the THz range. We would like to leave the statement of the Reviewer 3: "a major problem of the manuscript looks to be a chapter in a thesis that is not clearly written for a broader applied physics audience" to the Editor, since the comments of the Reviewers 1 and 2 are exactly opposite.

Comment of the Reviewer 1:
"The manuscript is overall well written, and the experimental results are shown at the level of journal publication"

Comment of the Reviewer 2:
"Overall, this manuscript is well organized and written."

Major Points - Central to the paper is design of the rotational delay line and a static retro-reflector. That is clearly a unique and clever optical design. The authors claimed that the surface roughness of less than 10 nm do not contribution to intensity noise, is unclear and do not appear to be well supported. It is necessary to consider roughness here with wavelength should be include in the supplementary information. From Fig.2, there is clear ripples from the rotational scanning (4), (8). It is unclear if those intensity ripples are created due to alignment of the static retro reflector? or the machining of the mirrors. This should be better discussed.

We agree with the Reviewer 3 that the core and the uniqueness of the rotational delay line is the combination of the static and rotating retro-reflectors and we thank her/him for the positive comments. Concerning Fig. 2, please take a look at the comments of Reviewer 1, subitem 5. We would like to provide data to support our statement that the surface quality of the rotating mirror does not add additional noise due to surface roughness. The figure below shows a standard deviation of the mean of four beams, two static beams and two Doppler shifted beams an the sample and normalization detector. The Doppler/static beams were recorded by blocking the static/Doppler beams. The standard deviation of static 1 and Doppler 2 beam are constant and do not change as a function of time and hence rota-

tion angle. The same is valid for static 2 and Doppler 1 beams. This directly shows that STD is the same for Doppler shifted and static beam. The difference in the STD of the beam is due to the power difference of the beams on the detector. Hence, we can be sure that surface roughness quality (¡ 10nm Ra) achieved with a diamond turning is sufficient for our applications. This discussion has now been added to the supplementary.

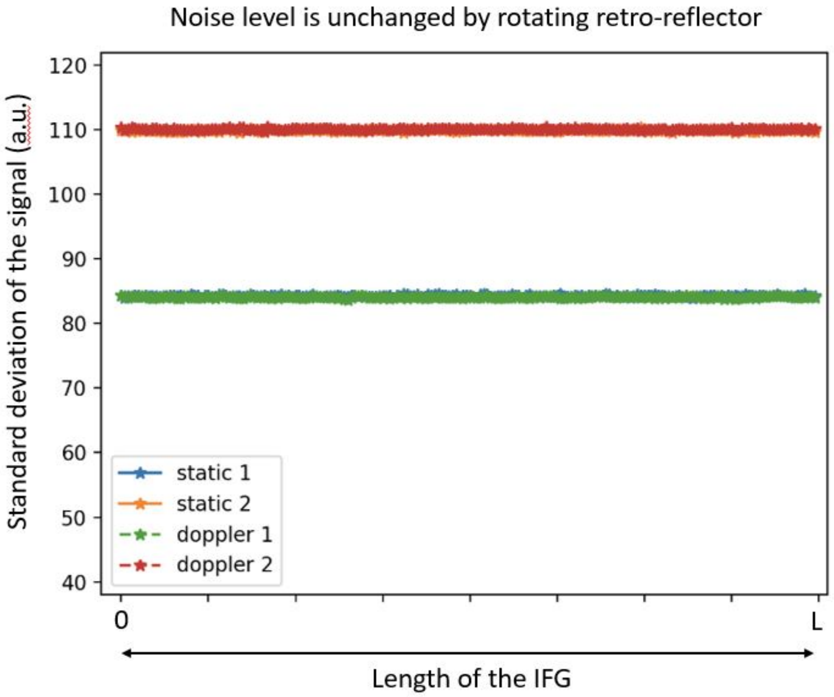

Figure 5: **surface roughness of the RDL**

- The authors tried to provide information of rotating delay line and beam entrance. There is not discussion on divergence and beam waist over multiple reflectors. This is likely to be a fairly challenging steps that does not appear to be discussed clearly. Alignment protocol for such a unique mirror scanning method would require significant alignment steps. For that, it will be greatly helpful for the authors to include so that readers are aware of the limitations here in beam waist and scanning angle. This is particularly important because clipping beam waist onto each scanning mirror will create poor reflectance.

The only requirement for the system is a collimated beam over 1 m in length with the diameter below 7mm in the delay line. Such conditions are relatively easy to achieve at the wavelength of 8 um. However, the beam diameter of the system can be increase by designing a rotating retro-reflector with different height. The alignment of the system is relatively straight forward, since the entire principle is based on retro-reflectors, which either reflect back the beam by shifting it in the plane (rotating retro-reflector) or lifting the beam in height and reflecting it back (static retro-reflector). We would like also to note that the polarisation of the system in preserved. We have now added a section about the beam waist and it influence on the total optical path length in the Supplementary Information.

- Section 3 of the paper talks about methane detection. While this is a good proof of concept, the discussion on the results do not clearly assert the sub-GHz resolution against current THz spectroscopy system. This lack of the discussion will proof difficult for readers to assess the advantage of such a rotational delay line.

In this case we disagree with the Reviewer, since our statement in the manuscript: "Providing sub-GHz spectral resolution shows a clear advantage of our approach, since such a high resolution is challenging to achieve with the sate-of-the-art FT spectrometers which are typically on the order of 0.2 cm1 (6 GHz) or 0.06 cm1 (1.8 GHz) for high resolution spectroscopy" clearly quantifies the advantages of our system.

Minor comments - There are generally a lack of attention on how the novelty of the rotational delay line has been presented. While the author shows that such a delay line can be useful, it rather unclear how they have clearly convincingly shown to surpass speed current THz spectroscopy system for both resolution and speed. The author keep mentioning about state of the art. But it is unclear what that actually is?

We thank the Reviewer for the comment. First of all, we would like to stress out that the proof-of-concept was demonstrated in the Mid-IR region (at 8 um wavelength) and not in the THz range. With this work we highlight the fact that dual-comb spectroscopy with a single frequency comb is possible thanks to the Doppler effect induced by the rotational retro-reflector. It is not the aim of the manuscript to outperform existing technology in speed or resolution, at this stage. As pointed out in the discussion section our demonstrated method can not only be used for real-time spectroscopy with a single frequency comb, but the system can be also modified and used for frequency comb ptychoscopy [31] and photo-acoustic dual-comb spectroscopy [36]. We

have now added the state-of-the-art specification of rapid scan FTIR in the manuscript and compare them to our system.

- In general, the paragraph are lengthy and feel more like a quick submission from a cut in a thesis page. Sentences do not provide clear concise discussions necessary in a research article. 1) Long sentences can be cut down significantly for example "Providing sub-GHz spectral resolution shows a clear advantage of our approach, since such a high resolution is challenging to achieve with the state-of-the-art FT spectrometers which are typically on the order of 0.2 cm1 (6 GHz) or 0.06 cm1 (1.8 GHz) for high resolution spectroscopy". Could be rephrase as: Our rotational delay line achieve resolution of on the order of 0.2 cm1 (6 GHz) or 0.06 cm1 (1.8 GHz) close to the state of the art FT spectrometers.

We respectfully but strongly disagree with the Reviewer 3 statement: "In general, the paragraph are lengthy and feel more like a quick submission from a cut in a thesis page". This paper is not part of any PhD thesis. We also disagree with the above rephrasing, since it completely changes the meaning of the sentence. We have rephrased the above sentence and make it more clearer in the manuscript.

Reviewers' comments:

Reviewer #1 (Remarks to the Author):

There are incorrect claims in the authors' rebuttal report, which are discussed below. Before giving point-by-point comments, I summarize my review. The significance of this paper is the development of a new rapid rotational delay line potentially working for broadband spectral range. I suggest the authors focus on this point and tone down the other claims. There are many misleading points in the manuscript.

The manuscript should contain more realistic discussions on the broadband FTIR. For example, if one wants to demonstrate extremely broadband FTIR, one must make averaging the interferograms or spectra because of the trade-off relation between the SNR and spectral bandwidth. To take advantage of the rapid-scan measurement, one has to select a narrow bandwidth anyway. The only advantage of the authors' system to me is an easy switch of the measurement wavelength range only by changing the wavelength of the light source, which is not the case with the system with dispersive elements because one has to optimize these elements when changing the wavelength range. I would strongly suggest the authors make the significance clearer and reorganize the manuscript. The title of the manuscript, "Frequency chirped Fourier-transform spectroscopy," does not reflect the significance of the paper. I would suggest a title such as "Fourier-transform spectroscopy with a rapid-scan rotational broadband delay scanner".

I raise point-by-point replies to the authors' rebuttal comments below.

0. There are incorrect descriptions.
0-1. The authors mention in the manuscript that "However, these newly developed methods rely on dispersive elements such as gratings, dispersive fibers, or prisms. Hence, they can be operated only over a limited optical bandwidth, mainly at visible and near-infrared frequencies." I am disappointed that the authors did not modify their manuscript in a proper way. As I suggested in the previous review report, there have already been demonstrations in the mid-infrared region, and this is what the authors are also aware of. Why do the authors still exclude the previous mid-infrared demonstrations? The reference [14] is operated in the mid-infrared, and the paper [Hashimoto et al., Laser and Photonics Reviews 15, 2000374 (2021)] is also operated in the mid-infrared. Why do the authors ignore these?
0-2. The authors mention in the rebuttal report that "It is true that the system in [13] is much faster than our demonstration. However, this is due to the use of a polygonal mirror which at the same time severely limits the spectral resolution to the degree where the frequency comb modes cannot be resolved." In my understanding, this claim is wrong. I carefully checked ref [13] and found that they achieved a spectral resolution of 11.5 GHz over 1.5 THz at a scan rate of 12 kHz (See Figure 5 in ref [13]). This resolution and bandwidth are actually comparable to the authors' demonstration. Since the authors' QCL comb's mode-spacing is 19.7 GHz, it can also be resolved with their system.

1. The authors mention that "Gratings of limited bandwidth are of course available across a wide spectral region. However, a continuous coverage from optical frequencies down to THz is challenging to achieve with a single dispersive element as used in Ref [13-16] and the two references mentioned above." I agree that the authors' delay scanner without dispersive elements could support an extremely broadband spectral region, but if the broadband feature is claimed in the context of FTIR, a discussion on SNR must be made. As I mentioned above, one has to sacrifice the measurement speed and average interferograms or spectra. In this context, when does the rapid broadband delay scanner useful? One can use a conventional slower linear scanner if averaging is required anyway. The current manuscript is not convincing enough from a realistic point of view.

2. The authors mention that "By discussing about the broad band range we imply a bandwidth typical for an FTIR from 1 micrometer (299.8 THz) to 20 micrometers (14.9 THz). We are not aware of any

dispersive element which could cover continuously the above mentioned bandwidth. We have now stated the discussed band-width in the manuscript." Same comment as above. A discussion on SNR is missing if the authors claim such a broadband spectrum measurement.

3. The authors mention that "The spectral resolution in our system is constant and is given by the total optical path length of the rotational delay line. The acquisition time for the IFG can be adjusted and is only limited by the motor angular velocity. This is different to the state-of-the-art FTIR with the linear delay line." I think the "decoupling" thing the authors insist on is nothing new, which is seen in many systems. It is just coming from the fact that the optical delay is prefixed. The conventional linear delay line can also be used as the "prefixed delay" mode, and it is not different from the author's system. I would suggest the authors make tone down on the "decoupling" claims.

4. I am disappointed that the authors did not show the requested linearity data of the detector. It is normal that detectors do not work in a linear regime up to the saturation power. I strongly request the authors show the linearity data because it is important to discuss the SNR of the measurement, which, in the end, is primarily related to the significance of this work.

6. I suggest the author add the visibility data, perhaps in Supplementary information.

Reviewer #2 (Remarks to the Author):

The authors have addressed my concerns properly. I recommend to publish this paper as it is.

Reviewer #3 (Remarks to the Author):

In the revised manuscript, I am pleased that the author has taken on board some of the comments and providing clarification to each comment. Markmann and co-workers have included additional information on mirror surfaces roughness that will prove critical to evaluating the reflecting efficiency of the mirrors (supplementary figure), beam waist and the total optical path. However, I find that the general writing style can be improved to improve concise discussed.
For example,
"Providing sub-GHz spectral resolution shows a clear advantage of our system with respect to the state-of-the-art FT spectrometer, which achieves a typical resolution of 0.06 cm-1 (1.8 GHz) with a much larger footprint. Also ms acquisition time with 0.5 cm-1 (20 GHz) spectral resolution is not possible to achieve for rapid scan FT spectrometers. The typical spectral resolution for 10 ms acquisition time would be larger than 25 cm-1, hence the laser modes would not be resolved."

We demonstrated that sub-GHz spectral resolution displays a technical advantage over the state-of-the-art FT spectrometer that can only achieve a typical resolution of 0.06 cm-1 (1.8 GHz) only with with a much larger footprint. More importantly, we demonstrated millisecond acquisition time with 0.5 cm-1 (20 GHz) spectral resolution that is not possible using existing rapid scan FT spectrometers (~25 cm-1 and The typical spectral resolution for 10 ms acquisition time) that is insufficient to resolve laser modes.

There are many sentences in the manuscript that can be significantly improve to raise the readability of the manuscript.

That said, the technical merits of revised manuscript especially with the inclusion of the mirror and beam alignment details warrant acceptance. However, the readability of the manuscript can be improved significantly.

Rebuttal letter for paper COMMSPHYS-22-0307A

We would like to thank all the thee Reviewers for re-examining our manuscript and giving their advices.Here below we detail the answers. All changes in the main manuscript are highlighted with GREEN color .The supplementary material has been updated and two new figures have been inserted with linearity data of the detector and fringe visibility of the interferogram.

Giacomo Scalari
Sergej Markmann

Answers to Reviewer 1

Reviewer 1 (Remarks to the Author):

There are incorrect claims in the authorsx2019; rebuttal report, which are discussed below. Before giving point-by-point comments, I summarize my review. The significance of this paper is the development of a new rapid rotational delay line potentially working for broadband spectral range. I suggest the authors focus on this point and tone down the other claims. There are many misleading points in the manuscript.

The manuscript should contain more realistic discussions on the broadband FTIR. For example, if one wants to demonstrate extremely broadband FTIR, one must make averaging the interferograms or spectra because of the trade-off relation between the SNR and spectral bandwidth. To take advantage of the rapid-scan measurement, one has to select a narrow bandwidth anyway. The only advantage of the authors' system to me is an easy switch of the measurement wavelength range only by changing the wavelength of the light source, which is not the case with the system with dispersive elements because one has to optimize these elements when changing the wavelength range. I would strongly suggest the authors make the significance clearer and reorganize the manuscript. The title of the manuscript, "Frequency chirped Fourier-transform spectroscopy" does not reflect the significance of the paper. I would suggest a title such as "Fourier-transform spectroscopy with a rapid-scan rotational broadband delay scanner"

I raise point-by-point replies to the authors' rebuttal comments below.

0. There are incorrect descriptions. 0-1. The authors mention in the manuscript that "However, these newly developed methods rely on dispersive

elements such as gratings, dispersive fibers, or prisms. Hence, they can be operated only over a limited optical bandwidth, mainly at visible and near-infrared frequencies".

I am disappointed that the authors did not modify their manuscript in a proper way. As I suggested in the previous review report, there have already been demonstrations in the mid-infrared region, and this is what the authors are also aware of. Why do the authors still exclude the previous mid-infrared demonstrations? The reference [14] is operated in the mid-infrared, and the paper [Hashimoto et al., Laser and Photonics Reviews 15, 2000374 (2021)] is also operated in the mid-infrared. Why do the authors ignore these?

We thank the Reviewer for the careful re-reading of our manuscript and the suggestions about the content and the literature. We did not ignore the mid-IR demonstrations, we now modified the text to make it more clear. We do not ignore as well the reference 14, since it is already contained in our bibliography. The very interesting and fast method presented in Ref.14 is indeed based on dispersive elements, somewhat restring its applicability over a broad bandwidth, as also for Hashimoto LPR 2021 paper, that we also added to our reference list since it is highly relevant.

0-2. The authors mention in the rebuttal report that "It is true that the system in [13] is much faster than our demonstration. However, this is due to the use of a polygonal mirror which at the same time severely limits the spectral resolution to the degree where the frequency comb modes cannot be resolved"

In my understanding, this claim is wrong. I carefully checked ref [13] and found that they achieved a spectral resolution of 11.5 GHz over 1.5 THz at a scan rate of 12 kHz (See Figure 5 in ref [13]). This resolution and bandwidth are actually comparable to the authors' demonstration. Since the authors' QCL comb's mode-spacing is 19.7 GHz, it can also be resolved with their system.

We thank the Reviewer for checking the reference and we agree that indeed the resolution is the same and the speed is higher. As discussed also lower, we include these references in our manuscript and moreover we make no claim in the text that our system is the fastest and has the highest resolution.

1. The authors mention that " Gratings of limited bandwidth are of course available across a wide spectral region. However, a continuous coverage from optical frequencies down to THz is challenging to achieve with a single dispersive element as used in Ref [13-16] and the two references

mentioned above" I agree that the authors' delay scanner without dispersive elements could support an extremely broadband spectral region, but if the broadband feature is claimed in the context of FTIR, a discussion on SNR must be made. As I mentioned above, one has to sacrifice the measurement speed and average interferograms or spectra. In this context, when does the rapid broadband delay scanner useful? One can use a conventional slower linear scanner if averaging is required anyway. The current manuscript is not convincing enough from a realistic point of view.

We respectfully disagree with the Reviewer's statement that our manuscript is "not convincing enough from a realistic point of view". We present experimental data where we performed different spectral measurements employing "realistic" conditions. The possibility to perform rapid (sub-second) spectral measurements at resolutions below 1 cm^{-1} is highly relevant for a number of applications (chemical monitoring,protein dynamics studies, micromachining). that can be for sure classified as "realistic". Moreover, we would like to point out that the above mentioned application typically employ multiple laser sources with limited bandwidth in the spectral regions of interest, which can be located far apart from each other. In this way the SNR can be kept high by maintaining high acquisition speed. This is the reason why it is important that the system supports large optical bandwidth.

2. The authors mention that "By discussing about the broad band range we imply a bandwidth typical for an FTIR from 1 micrometer (299.8 THz) to 20 micrometers (14.9 THz). We are not aware of any dispersive element which could cover continuously the above mentioned bandwidth. We have now stated the discussed band-width in the manuscript" Same comment as above. A discussion on SNR is missing if the authors claim such a broadband spectrum measurement.

We would like to underline that we do not claim the presence of a broadband spectral measurement in the paper. Our statement is relative to the potential extension of the demonstrated technique to a broader bandwidth since there are no intrinsic limitation due to the presence of dispersive optical elements. In this respect we modified the abstract of the paper. A detailed SNR discussion is carried out on the specific experimental implementation and a general SNR discussion including broadband sources is in our opinion outside the scope of the present paper.

3. The authors mention that "The spectral resolution in our system is constant and is given by the total optical path length of the rotational delay line. The acquisition time for the IFG can be adjusted and is only limited

by the motor angular velocity. This is different to the state-of-the-art FTIR with the linear delay line.” I think the ”decoupling” thing the authors insist on is nothing new, which is seen in many systems. It is just coming from the fact that the optical delay is prefixed. The conventional linear delay line can also be used as the ”prefixed delay”; mode, and it is not different from the author’s system. I would suggest the authors make tone down on the decoupling claims.

We thank the Reviewer for this comment. What we are stating is that, since in the ”conventional” linear delay the mirror has to be linearly accelerated and decelerated (i.e. the center of mass has a linear motion) , there is an intrinsic limitation on the speed at which this movement can be performed keeping the same delay length (mirror dimensions and mass and beam size play a big role). This limit can be pushed much further with the rotational delay approach since there is no linear motion of the center of mass of the mirror. This translates into an effective decoupling of the resolution from the acquisition speed.

4. I am disappointed that the authors did not show the requested linearity data of the detector. It is normal that detectors do not work in a linear regime up to the saturation power. I strongly request the authors show the linearity data because it is important to discuss the SNR of the measurement, which, in the end, is primarily related to the significance of this work.

As requested by the Reviewer, we provide the linearity data of the employed detector here below in Fig.1, and we added it as well in the Supplementary Information.

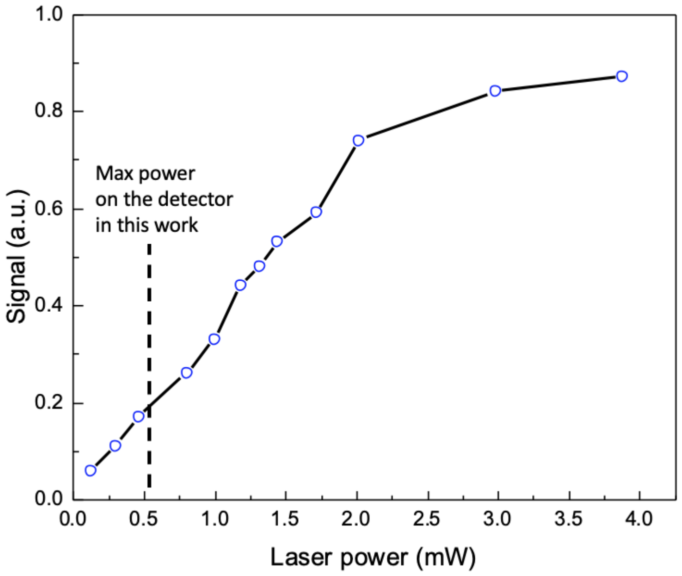

Figure 1: **Response of the employed photodetecto with indicated the laser power used in the experiments**

6. I suggest the author add the visibility data, perhaps in Supplementary information.

We thanks the reviewer for this suggestion. We have now added this data to the supplementary.

Answers to Reviewer 2

Reviewer 2 (Remarks to the Author):

The authors have addressed my concerns properly. I recommend to publish this paper as it is.

We thank Reviewer 2 for her/his appreciation of our corrections and for the indication about paper publication.

Answers to Reviewer 3

Reviewer 3 (Remarks to the Author):

In the revised manuscript, I am pleased that the author has taken on board some of the comments and providing clarification to each comment. Markmann and co-workers have included additional information on mirror surfaces roughness that will prove critical to evaluating the reflecting efficiency of the mirrors (supplementary figure), beam waist and the total optical path. However, I find that the general writing style can be improved to improve concise discussed. For example, "Providing sub-GHz spectral resolution shows a clear advantage of our system with respect to the state-of-the-art FT spectrometer, which achieves a typical resolution of 0.06 cm-1 (1.8 GHz) with a much larger footprint. Also ms acquisition time with 0.5 cm-1 (20 GHz) spectral resolution is not possible to achieve for rapid scan FT spectrometers. The typical spectral resolution for 10 ms acquisition time would be larger than 25 cm-1, hence the laser modes would not be resolved"

We demonstrated that sub-GHz spectral resolution displays a technical advantage over the state-of-the-art FT spectrometer that can only achieve a typical resolution of 0.06 cm-1 (1.8 GHz) only with with a much larger footprint. More importantly, we demonstrated millisecond acquisition time with 0.5 cm-1 (20 GHz) spectral resolution that is not possible using existing rapid scan FT spectrometers (25 cm-1 and The typical spectral resolution for 10 ms acquisition time) that is insufficient to resolve laser modes.

There are many sentences in the manuscript that can be significantly improve to raise the readability of the manuscript.

That said, the technical merits of revised manuscript especially with the inclusion of the mirror and beam alignment details warrant acceptance. However, the readability of the manuscript can be improved significantly.

We thank Reviewer 3 for her/his comments and suggestions about the readability of the manuscript. We rewrote and corrected several parts following these indications, as visible from the new version of the manuscript with highlighted changed parts.

REVIEWERS' COMMENTS:

Reviewer #3 (Remarks to the Author):

accept as it is
